# CMA-associated cellular state remodeling defines prognostic heterogeneity and identifies ARRDC3 as a protective functional gene in small cell lung cancer

**DOI:** 10.3389/fcell.2026.1894699

**Published:** 2026-07-17

**Authors:** Jiahong Han, Shun Xu, Yunfeng Jiang

**Affiliations:** 1 Department of Thoracic Surgery, Shenyang Fifth People’s Hospital, Shenyang, China; 2 Department of Thoracic Surgery, The First Hospital of China Medical University, Shenyang, China; 3 Department of Thoracic Surgery, Yantai Yuhuangding Hospital, Yantai, China

**Keywords:** ARRDC3, chaperone-mediated autophagy, drug sensitivity, machine learning, prognostic signature, single-cell RNA sequencing, small cell lung cancer

## Abstract

**Background:**

Small cell lung cancer (SCLC) is a highly aggressive malignancy characterized by rapid progression, early metastasis, frequent relapse, and treatment resistance. Although chemoimmunotherapy has improved outcomes in a subset of patients, reliable molecular stratification tools reflecting tumor heterogeneity and survival risk remain limited. Chaperone-mediated autophagy (CMA) is involved in proteostasis, metabolic adaptation, and stress responses, but its cellular heterogeneity and prognostic relevance in SCLC remain unclear.

**Methods:**

This study integrated single-cell RNA sequencing data, bulk transcriptomic cohorts, and clinical information to characterize CMA-related heterogeneity in SCLC. At the single-cell level, SCLC subtypes were annotated, CMA scores were calculated, and differences among baseline, sensitive, and resistant states were evaluated. At the bulk level, tumor versus normal differential expression analysis and weighted gene co-expression network analysis were performed to identify CMA-related candidate genes. A CMA-related Risk Score was constructed through comprehensive machine learning comparison and evaluated using survival analysis, time-dependent receiver operating characteristic curves, Cox regression, nomogram analysis, functional enrichment, immune microenvironment analysis, and drug sensitivity prediction. ARRDC3 was further validated by *in vitro* experiments.

**Results:**

Single-cell analysis revealed marked SCLC subtype heterogeneity and nonuniform CMA activity across cellular subtypes. Significant CMA score differences between sensitive and resistant cells were observed in the SCLC-A_NR0B1/MYCL+ and SCLC_Hypoxia_glycolytic subtypes. Integration of CMA-related co-expression modules with tumor-associated differentially expressed genes identified 53 candidate genes. The machine learning-derived Risk Score effectively stratified patients into high-risk and low-risk groups across the combined cohort, GSE60052 cohort, and cBioPortal cohort, with high-risk patients showing significantly poorer overall survival. The Risk Score remained an independent prognostic factor and was associated with proliferative, cell-cycle, metabolic, immune, and drug sensitivity-related features. *In vitro* experiments showed that ARRDC3 overexpression suppressed proliferation, colony formation, migration, and invasion in H446 cells.

**Conclusion:**

This study revealed CMA-related cellular heterogeneity in SCLC and established a robust prognostic Risk Score associated with survival, biological pathway activity, immune microenvironment features, and potential therapeutic responses. Functional validation of ARRDC3 further supports the biological relevance of this model, providing new insights into SCLC molecular risk stratification and CMA-associated resistance states.

## 1 Introduction

Small cell lung cancer (SCLC) is one of the most aggressive histological subtypes of lung cancer and is characterized by rapid tumor growth, early metastatic dissemination, frequent post-treatment relapse, and poor overall prognosis (Rudin et al., 2021). Most patients are diagnosed at an extensive stage, and platinum-based chemotherapy combined with etoposide has long remained the standard first-line treatment (Ganti et al., 2021). In recent years, the introduction of immune checkpoint inhibitors in combination with chemotherapy has provided modest survival benefits for patients with extensive-stage SCLC and has established chemoimmunotherapy as an important first-line therapeutic strategy (Horn et al., 2018; Paz-Ares et al., 2019). However, the overall clinical benefit remains limited, as a substantial proportion of patients exhibit poor initial responses and most eventually develop acquired resistance and disease progression (Liu et al., 2021). Moreover, SCLC is increasingly recognized as a biologically heterogeneous disease characterized by substantial transcriptional diversity and cellular plasticity (Chan et al., 2021; Tian et al., 2022). Previous studies have proposed molecular classification systems based on key transcription factors, including achaete-scute homolog 1 (ASCL1), neurogenic differentiation factor 1 (NEUROD1), POU class 2 homeobox 3 (POU2F3), and yes-associated protein 1 (YAP1), demonstrating that distinct SCLC subtypes differ in neuroendocrine differentiation, proliferative activity, inflammatory phenotypes, therapeutic responses, and clinical outcomes (Baine et al., 2020; Qi et al., 2022). Therefore, identifying molecular features associated with tumor heterogeneity, therapeutic resistance, and prognostic variation is of considerable importance for improving risk stratification and developing novel therapeutic strategies for SCLC.

Chaperone-mediated autophagy (CMA) is a selective lysosomal degradation pathway in which substrate proteins containing KFERQ-like motifs are recognized by molecular chaperones and subsequently translocated into the lysosomal lumen through lysosome-associated membrane protein type 2A (LAMP2A)-mediated transport (Dice, 1990; Cuervo and Dice, 1996). Unlike macroautophagy, CMA exhibits strong substrate specificity and plays important roles in protein quality control, cellular stress adaptation, metabolic homeostasis, and oxidative stress responses (Kaushik and Cuervo, 2018; Wang et al., 2025). Increasing evidence suggests that CMA exerts multifaceted effects during tumor initiation and progression by regulating proteostasis, metabolic adaptation, and cellular survival under therapeutic stress (Liu et al., 2023; Teixeira et al., 2024; Ichikawa et al., 2020). This biological function may be particularly relevant to SCLC, a malignancy characterized by rapid proliferation, high metabolic demand, initial sensitivity to cytotoxic therapy followed by frequent relapse, and pronounced cellular plasticity (Gazdar et al., 2017; Shi et al., 2023). Under therapeutic pressure, SCLC cells may need to maintain protein quality control, remove damaged or misfolded proteins, and adjust metabolic programs to survive cytotoxic stress (Chen X. et al., 2023). As a selective lysosomal degradation pathway involved in proteostasis maintenance, stress responses, and metabolic regulation, CMA may participate in the maintenance of stress-adapted and treatment-resistant cellular states (Zhang et al., 2025). Consistent with this rationale, CMA-associated molecular programs may contribute to cellular state remodeling during therapeutic challenge (Shi et al., 2023; Hosaka et al., 2021). However, the distribution of CMA activity across distinct SCLC cellular subtypes, its relationship with treatment-sensitive and treatment-resistant states, and the prognostic value of CMA-associated transcriptional features remain largely unexplored.

Conventional bulk transcriptomic analyses can leverage large sample sizes and long-term clinical follow-up data to identify prognostic biomarkers, but they are inherently limited in their ability to resolve the contributions of distinct cellular states (Ysebaert et al., 2021). In contrast, single-cell RNA sequencing (scRNA-seq) enables the characterization of intratumoral heterogeneity at cellular resolution but is often constrained by limited clinical outcome information (Kuksin et al., 2021). Integrating scRNA-seq and bulk transcriptomic data provides an opportunity to bridge cellular-state characterization with clinical prognostic assessment (Feng et al., 2023). In parallel, machine learning approaches have emerged as powerful tools for feature selection and prognostic model development (Pudova et al., 2025). Systematic evaluation of multiple algorithm combinations across independent cohorts can improve model robustness and enhance translational potential (Xie et al., 2025). Therefore, an integrative framework combining single-cell heterogeneity analysis, bulk transcriptomic co-expression networks, and machine learning-based modeling may provide new insights into prognostic stratification and resistance-associated molecular states in SCLC.

In the present study, we integrated scRNA-seq data, bulk transcriptomic profiles, and clinical follow-up information to systematically investigate CMA-associated heterogeneity and its prognostic significance in SCLC. We first characterized the distribution of CMA activity and subtype composition across different treatment-response states at the single-cell level. Subsequently, bulk tumor versus normal differential expression analysis, weighted gene co-expression network analysis (WGCNA), and machine learning strategies were integrated to identify CMA-related candidate genes and construct a CMA-related prognostic Risk Score, which was further evaluated across multiple independent cohorts. Functional pathway analysis, immune microenvironment characterization, and drug sensitivity prediction were performed to explore the biological features associated with the model. Finally, *in vitro* experiments were conducted to validate the functional role of ARRDC3, a key gene identified by the model, in regulating malignant phenotypes of SCLC cells. This study aimed to establish a robust CMA-related molecular stratification model and provide new insights into resistance-associated cellular states and potential therapeutic targets in SCLC.

## 2 Materials and methods

### 2.1 Data acquisition and preprocessing

This study integrated scRNA-seq data and bulk transcriptomic data to systematically evaluate CMA-related characteristics and their prognostic value in SCLC. The scRNA-seq dataset was obtained from the Gene Expression Omnibus (GEO) database under the accession number GSE138267 and included 20 eligible samples. After merging the raw single-cell data, a total of 114,383 cells were obtained. Subsequently, 15,594 potential doublets were identified and removed using scDblFinder, leaving 98,789 cells. After further quality control filtering, 66,405 high-quality cells were retained for downstream single-cell analyses.

Bulk transcriptomic data were obtained from the George cohort and the GSE60052 cohort. The George cohort, also referred to as the cBioPortal SCLC cohort in this study, was derived from the study “Comprehensive genomic profiles of small cell lung cancer” and included 73 samples with complete survival information. The GSE60052 cohort was derived from the study “Genomic Landscape Survey Identifies SRSF1 as a Key Oncodriver in Small Cell Lung Cancer” and included 79 SCLC tumor samples and 7 normal control samples, among which 45 tumor samples had available survival information. The GSE60052 cohort was used for tumor versus normal differential expression analysis, whereas samples with survival information from the George and GSE60052 cohorts were used for prognostic model construction, validation, and downstream clinical analyses.

For bulk data preprocessing, common genes shared by the George and GSE60052 cohorts were first extracted. According to the distribution of expression values, the GSE60052 expression matrix was retained on its original log2-transformed scale, whereas the George cohort expression matrix was transformed using log2 (x + 1) to harmonize the expression scale. Infinite values were replaced with missing values, and missing values were imputed using the median expression value of the corresponding gene. Genes with extremely low variance were removed to avoid potential effects on batch correction and downstream modeling. The two bulk transcriptomic datasets were then merged, and batch effects were corrected using the ComBat function from the sva package (Johnson et al., 2007). Principal component analysis (PCA) was performed to evaluate sample distribution before and after batch-effect correction. Samples included in prognostic model analyses were required to have complete overall survival (OS) time, survival status, and corresponding gene expression information.

### 2.2 Single-cell RNA-seq analysis

The scRNA-seq data were processed using Seurat (Stuart et al., 2019). Raw expression matrices were read according to their data formats and used to construct Seurat objects. To ensure data quality, the number of detected genes, unique molecular identifiers, mitochondrial gene percentage, ribosomal gene percentage, and hemoglobin gene percentage were calculated for each cell. Potential doublets were identified and removed using scDblFinder. Cells were then filtered according to quality control criteria, including nFeature_RNA between 500 and 7,500, nCount_RNA between 1,000 and 60,000, percent.mt ≤ 20%, percent.rb ≤ 50%, and percent.HB ≤ 5%.

After quality control, cells were normalized using the LogNormalize method with a scale factor of 10,000. The top 3,000 highly variable genes were identified using the vst method. During data scaling, mitochondrial gene percentage and unique molecular identifier counts were regressed out as potential confounding factors. PCA was then performed, and Harmony was used to correct batch effects associated with patient and sample_folder based on the first 30 principal components (Korsunsky et al., 2019). Harmony-corrected embeddings were used for nearest-neighbor graph construction, Uniform Manifold Approximation and Projection (UMAP) dimensionality reduction, and clustering analysis. Multiple clustering resolutions were evaluated, and the clustering result at resolution = 0.5 was used for subsequent cell subtype annotation. SCLC subtype annotation was performed based on canonical SCLC subtype markers and functionally relevant gene expression patterns. Cluster marker genes were identified using the FindAllMarkers function in Seurat with the Wilcoxon rank-sum test, using min.pct = 0.25 and logfc.threshold = 0.25.

To evaluate CMA activity at the single-cell level, a CMA score was calculated for each cell based on a recently reported CMA transcriptional network (Khawaja et al., 2025). This CMA-related gene set consisted of 84 genes and included three functional categories: CMA effector genes, positive regulatory genes, and negative regulatory genes. CMA effector genes represent core components directly involved in CMA execution, including substrate recognition, chaperone-mediated delivery, lysosomal binding, and substrate translocation. Therefore, these genes were assigned a higher weight in the scoring formula to emphasize the contribution of core CMA machinery. Positive regulatory genes were added to the score, whereas negative regulatory genes were subtracted to reflect their opposite regulatory effects on CMA activity. For each cell, the expression sums of these three gene categories were calculated, and the CMA score was defined as follows:

$$\text{CMA score}=\left(2\;\times \text{effector gene expression sum}\right.-\text{ negative regulator expression sum}+\text{positive regulator expression sum})/84.$$

The denominator, 84, represents the total number of genes included in the CMA-related gene set and was used for normalization across cells.

According to treatment-response information, cells were categorized into baseline, sensitive, and resistant states. Differences in SCLC subtype composition and CMA scores across treatment-response states were then evaluated. Comparisons between two groups in the overall SCLC cell population and within individual SCLC subtypes were performed using the Wilcoxon rank-sum test. For SCLC subtypes showing significant differences in CMA scores, the FindMarkers function was used to compare differential expression between resistant and sensitive cells, with the Wilcoxon rank-sum test used for statistical analysis. To reduce the influence of lowly expressed genes and minor expression changes, only genes expressed in at least 10% of cells and with an average log2 fold change greater than 0.25 were retained for subsequent analyses.

### 2.3 Differential expression analysis and WGCNA

To identify differentially expressed features between SCLC tumor and normal tissues, tumor versus normal differential expression analysis was performed using the GSE60052 cohort. This cohort included 79 SCLC tumor samples and 7 normal control samples. Before analysis, the expression matrix was processed for gene annotation, sample group matching, and missing value handling. Differential expression analysis was performed using the limma package (Ritchie et al., 2015). A design matrix and a tumor versus normal contrast matrix were first constructed, followed by empirical Bayes moderation to estimate differential expression statistics. Differentially expressed genes were defined using the criteria of adjusted P value < 0.05 and |log2 fold change| > 1. Genes with log2 fold change > 1 were considered upregulated, whereas genes with log2 fold change < −1 were considered downregulated. Differential expression results were visualized using a volcano plot, and representative upregulated and downregulated genes were displayed in a heatmap. For heatmap visualization, expression values were Z-score standardized by gene to show expression pattern differences between tumor and normal samples.

To further identify co-expression gene modules associated with CMA activity, WGCNA was performed (Langfelder and Horvath, 2008). First, the CMA score of each bulk sample was calculated based on the CMA-related gene set using the single-sample gene set enrichment analysis (ssGSEA) method implemented in the GSVA package, with method = “ssgsea,” kcdf = “Gaussian,” and abs.ranking = TRUE. The expression matrix was then matched with sample-level CMA scores, and genes with zero expression variance were removed. The goodSamplesGenes function was used to assess sample and gene quality, and sample clustering was performed to evaluate the presence of obvious outliers.

During co-expression network construction, the pickSoftThreshold function was used to select an appropriate soft-thresholding power from candidate soft powers to satisfy the scale-free topology criterion. An unsigned co-expression network was then constructed using the blockwiseModules function. Co-expression modules were identified using the dynamic tree cut method, with the minimum module size set to 50 and the module merging threshold set to 0.15. Module eigengenes were used to represent the overall expression pattern of each module. Pearson correlation coefficients between module eigengenes and CMA scores were calculated to identify modules associated with CMA activity. Because the aim of this analysis was to identify gene modules positively associated with increased CMA activity, modules showing significant positive correlations with the CMA score were defined as CMA-related key modules and used for subsequent candidate gene screening. Negatively correlated modules were not included in the downstream model construction because they reflected gene programs inversely associated with CMA activity and were not the primary focus of the present study.

Within key modules, module membership and gene significance were further calculated. Module membership represented the correlation between individual gene expression and the corresponding module eigengene, whereas gene significance represented the correlation between individual gene expression and the CMA score. The relationship between module membership and gene significance was analyzed to evaluate the consistency between genes within key modules and the CMA-related phenotype.

### 2.4 Identification and functional annotation of CMA-related candidate genes

The single-cell analysis was used to characterize CMA-related cellular heterogeneity and treatment-response-associated CMA activity across SCLC subtypes. Candidate gene screening for prognostic model construction was then performed at the bulk transcriptomic level. Specifically, differentially expressed genes from the GSE60052 tumor versus normal analysis were intersected with genes from WGCNA-derived CMA-related key modules. Genes from modules significantly positively correlated with the CMA score were merged as the CMA-related module gene set. The overlap between differentially expressed genes and CMA-related module genes was defined as CMA-related candidate genes and used for subsequent machine learning-based model construction.

To explore the potential biological functions of the candidate genes, Gene Ontology (GO) enrichment analysis was performed using the clusterProfiler package. GO analysis included biological process (BP), cellular component (CC), and molecular function (MF) categories. P values were adjusted using the Benjamini–Hochberg method. Enrichment results were ranked according to adjusted P values and visualized using bubble plots. Correlations among candidate genes were further assessed and visualized using a correlation heatmap to examine the expression relationships among genes retained for subsequent model construction.

### 2.5 Machine learning-based prognostic model construction

After obtaining CMA-related candidate genes, a prognostic model for SCLC was constructed based on candidate gene expression profiles and OS information. The combined cohort was used as the training cohort, and model performance was further evaluated in the GSE60052 and cBioPortal cohorts. To systematically compare the predictive performance of different modeling strategies, 101 machine learning algorithm combinations were evaluated, including random survival forest (RSF), StepCox, CoxBoost, elastic net, least absolute shrinkage and selection operator (LASSO), Ridge regression, gradient boosting machine (GBM), SuperPC, partial least squares Cox (PLS-Cox), survival support vector machine (survival-SVM), and Bayesian additive regression trees (BART).

Before model construction, expression values of all candidate genes were standardized. The RSF model was constructed using the randomForestSRC package, with survival outcome represented as Surv (OS.time, OS). The main parameters were set as ntree = 1,000, nodesize = 5, and splitrule = “logrank,” and variable importance was calculated. For combination models using RSF as the feature selection step, top-ranked candidate genes were first selected according to RSF variable importance and then incorporated into CoxBoost, elastic net, LASSO, Ridge, GBM, SuperPC, PLS-Cox, survival-SVM, or BART models. For CoxBoost models, the penalty parameter was estimated using optimCoxBoostPenalty, and the optimal number of boosting steps was determined using 10-fold cross-validation. Elastic net, LASSO, and Ridge models were implemented using glmnet, with optimal regularization parameters selected by cross-validation.

Model performance was assessed using the concordance index (C-index), which was calculated in the combined cohort and further evaluated separately in the GSE60052 and cBioPortal cohorts. For each algorithm combination, overall performance was summarized by considering the mean C-index across cohorts and the consistency of C-index values among different cohorts. The final model was selected according to both predictive performance and cross-cohort stability, rather than performance in a single cohort alone. In addition, model parsimony was considered to avoid unnecessary feature-selection steps that could increase the risk of overfitting. For the selected model, variable importance was further calculated to evaluate the relative contribution of each candidate gene to prognostic prediction.

The selected model was then used to calculate a Risk Score for each patient. Patients were stratified into high-risk and low-risk groups using the median Risk Score as the cutoff. Kaplan-Meier survival analysis was performed to compare OS between the two groups, and differences were assessed using the log-rank test. Time-dependent receiver operating characteristic (ROC) curves were generated to evaluate the predictive performance of the Risk Score for 1-, 3-, and 5-year OS.

### 2.6 Clinical and prognostic evaluation

To evaluate the clinical relevance and prognostic value of the Risk Score, clinical variables including age, sex, Union for International Cancer Control (UICC) stage, T stage, N stage, M stage, survival status, and risk group were integrated. Associations between categorical clinical variables and risk group were assessed using the chi-square test or Fisher’s exact test. Differences in Risk Score distribution among UICC stages were compared using nonparametric tests, with pairwise comparisons performed when appropriate. To explore the association between the Risk Score and disease stage status, patients were further classified into UICC stage I-II and UICC stage III-IV groups, and ROC curves were generated to calculate the area under the curve (AUC).

To further assess the prognostic stratification ability of the Risk Score across different clinical contexts, Kaplan-Meier survival analyses were performed in patients with UICC stage I-II disease, patients with UICC stage III-IV disease, patients aged ≤60 years, and patients aged >60 years. Differences in OS between high-risk and low-risk groups were evaluated using the log-rank test.

To determine whether the Risk Score had independent prognostic value, univariate and multivariate Cox proportional hazards regression analyses were performed. Before Cox regression analysis, selected clinical variables were regrouped as follows: T stage was classified as T1 + T2 versus T3 + T4, N stage was classified as N0 versus N1 + N2 + N3, and UICC stage was classified as I + II versus III + IV. Univariate Cox regression was used to assess associations between the Risk Score, clinical variables, and OS, whereas multivariate Cox regression was used to evaluate the independent prognostic effect of the Risk Score after adjustment for clinical variables. Results were reported as hazard ratios (HRs), 95% confidence intervals (95% CIs), and P values.

Based on the multivariate Cox regression model, a nomogram was constructed to predict 1-, 3-, and 5-year OS probabilities in patients with SCLC. The predictive performance of the nomogram was evaluated using calibration curves by comparing predicted survival probabilities with observed survival outcomes. Decision curve analysis (DCA) was further performed to assess the clinical net benefit of the nomogram across different threshold probabilities.

### 2.7 Functional enrichment analysis

To characterize functional pathway differences between high-risk and low-risk groups, gene set enrichment analysis was performed based on Hallmark gene sets. First, differential expression between high-risk and low-risk groups was evaluated, and genes were ranked according to differential expression statistics. Gene set enrichment analysis (GSEA) was then performed using the clusterProfiler package (Wu et al., 2021). GSEA results were evaluated based on normalized enrichment score (NES), P value, and adjusted P value.

In addition, the GSVA/ssGSEA method was used to calculate Hallmark pathway activity scores for each sample. Differences in pathway activity between high-risk and low-risk groups were then compared, and pathways were classified as activated, suppressed, or non-significant in the high-risk group according to the statistical results. Finally, the Risk Score was treated as a continuous variable and correlated with Hallmark pathway activity scores to evaluate the functional pathway states reflected by the Risk Score.

### 2.8 Immune microenvironment analysis

To evaluate the relationship between the CMA-related Risk Score and the tumor immune microenvironment, multiple immune infiltration analysis strategies were applied to systematically characterize immune features across different risk groups. First, the deconvo_tme function in the IOBR package was used to perform ESTIMATE analysis and calculate the Stromal Score, Immune Score, and ESTIMATE Score for each sample (Yoshihara et al., 2013). These scores were used to assess stromal components, immune infiltration levels, and tumor purity-related features. Differences in these scores between risk groups were compared using the Wilcoxon rank-sum test.

To further evaluate immune functional states across risk groups, ssGSEA implemented in the GSVA package was used to calculate immune function pathway activity scores based on predefined immune function gene sets. Differences in immune function scores between high-risk and low-risk groups were compared using the Wilcoxon rank-sum test, and multiple testing correction was performed using the Benjamini–Hochberg method.

In addition, ssGSEA was used to estimate the relative enrichment of immune cell subsets in each sample, including major immune cell types such as T cells, B cells, natural killer cells, macrophages, dendritic cells, and neutrophils. Differences in immune cell infiltration between high-risk and low-risk groups were then evaluated.

To further explore the relationship between model genes and immune cell infiltration, CIBERSORT implemented in the IOBR package was used to infer immune cell composition in each sample, with the number of permutations set to 100. Correlation analysis was then performed to assess associations between model gene expression and immune cell proportions, and the results were visualized using a correlation heatmap.

### 2.9 Drug sensitivity analysis and experimental validation

To assess potential differences in therapeutic responses between risk groups, drug sensitivity prediction analysis was performed based on the Genomics of Drug Sensitivity in Cancer (GDSC) database. Patients were first divided into high-risk and low-risk groups according to the median Risk Score. The oncoPredict package was then used to predict the half-maximal inhibitory concentration (IC50) of different drugs for each sample based on bulk transcriptomic profiles (Sebaugh, 2011). During drug sensitivity prediction, the training set was derived from the GDSC2 database, and the calculatePhenotype function was used to map expression profiles to drug response models. Predicted results were batch-corrected and standardized before downstream analysis.

A total of 10 representative antitumor agents were included in this study, including PF-4708671 targeting the PI3K/AKT/mTOR pathway, Trametinib targeting the MAPK/ERK pathway, receptor tyrosine kinase-related agents including Gefitinib, CEP-701, and Imatinib, the cell-cycle inhibitor PD-0332991, DNA damage response-related agents including AZD7762 and Veliparib, the epigenetic regulator Vorinostat, and the differentiation-inducing agent all-trans retinoic acid (ATRA). Differences in predicted IC50 values between risk groups were compared using the Wilcoxon rank-sum test and visualized using a bidirectional lollipop plot to show both the direction and statistical significance of drug sensitivity differences.

*In vitro* experiments were performed using the human SCLC cell line H446. H446 cells were cultured in medium supplemented with 10% fetal bovine serum (FBS) and 1% penicillin-streptomycin at 37 °C in a humidified incubator with 5% CO_2_. An ARRDC3 overexpression model was then established, with empty vector-transfected cells used as the negative control. ARRDC3 protein expression was verified by Western blot analysis.

Cell proliferation was assessed using the Cell Counting Kit-8 (CCK-8) assay. Transfected cells were seeded into 96-well plates, CCK-8 reagent was added at the indicated time points, and absorbance at 450 nm was measured. For the colony formation assay, transfected cells were seeded at low density into 6-well plates and cultured for approximately 10–14 days. Colonies were then fixed with paraformaldehyde, stained with crystal violet, and counted.

Cell migration and invasion were evaluated using wound healing and Transwell assays. For the wound healing assay, once cells reached appropriate confluence, a straight scratch was generated in the cell monolayer using a sterile pipette tip, and wound closure was recorded at 0 h and 24 h. For Transwell assays, migration assays were performed using chambers without Matrigel coating, whereas invasion assays were performed using chambers pre-coated with Matrigel. After incubation, cells that migrated or invaded through the membrane were fixed, stained, and counted under a microscope. All *in vitro* experiments were independently repeated at least three times. Experimental data are presented as mean ± standard deviation (SD), and differences between two groups were assessed using Student’s t-test or the Wilcoxon rank-sum test, with P < 0.05 considered statistically significant.

### 2.10 Statistical analysis

All statistical analyses were performed using R software version 4.4.2. Single-cell analyses were mainly performed using Seurat, Harmony, and scDblFinder. Differential expression analysis was conducted using limma. WGCNA was performed using the WGCNA package. Survival analyses were performed using survival and survminer. Time-dependent ROC curve analysis was performed using timeROC. Functional enrichment analysis was conducted using clusterProfiler and GSVA. Immune microenvironment analysis was performed using IOBR. Machine learning-based model construction was conducted using randomForestSRC and related machine learning frameworks.

For continuous variables, comparisons between two groups were performed using the Wilcoxon rank-sum test, whereas comparisons among multiple groups were performed using the Kruskal–Wallis test. Categorical variables were compared using the chi-square test or Fisher’s exact test. Correlation analysis was performed using Pearson correlation analysis. Differences between Kaplan-Meier survival curves were evaluated using the log-rank test. All statistical tests were two-sided, and P < 0.05 was considered statistically significant. Multiple testing correction was performed using the Benjamini–Hochberg method.

## 3 Results

**FIGURE 1:**
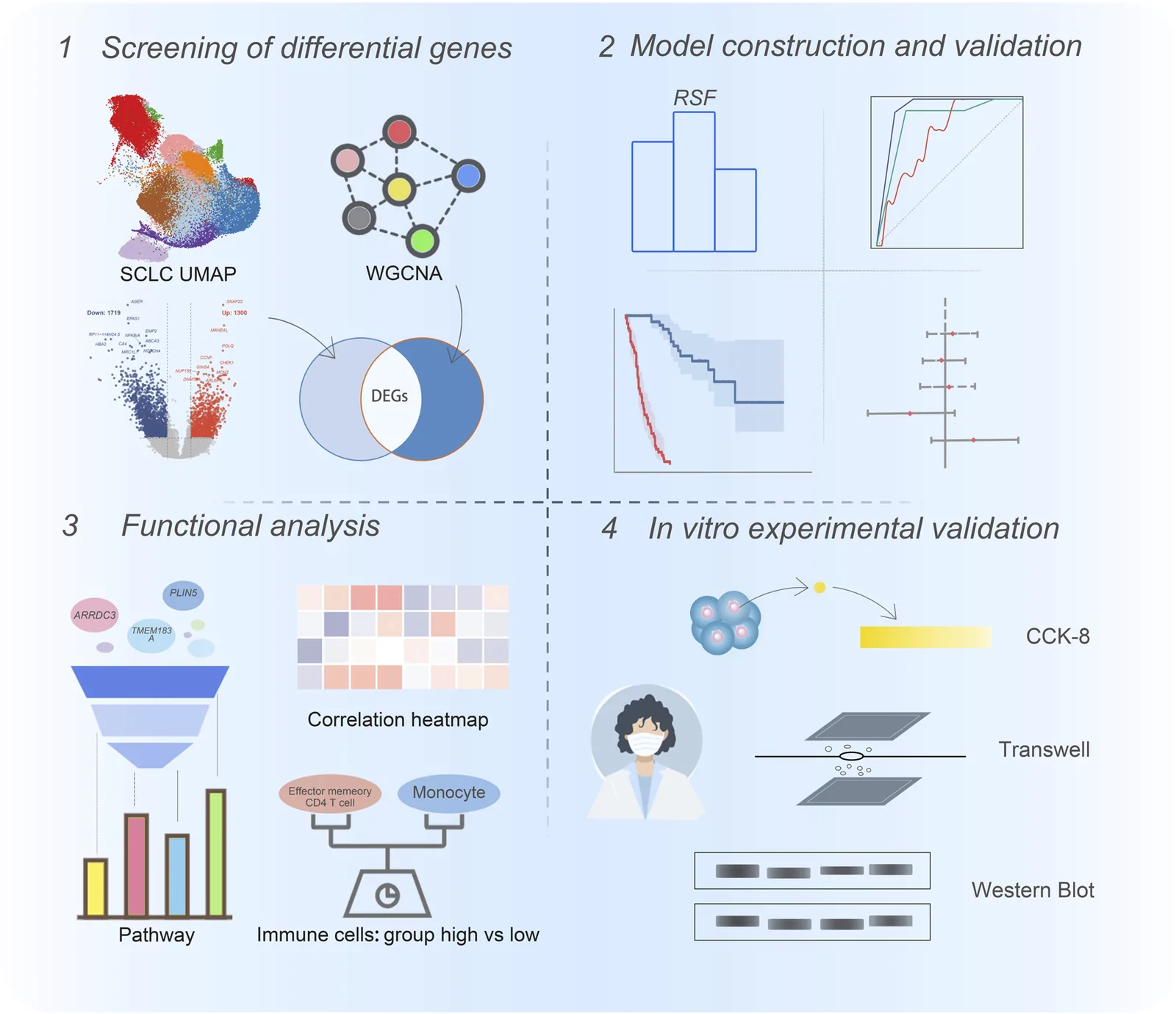
Schematic workflow of the study, including CMA-related candidate gene identification, machine learning-based prognostic model construction, functional and immune analyses, and experimental validation of ARRDC3 in SCLC cells.

The overall workflow of the present study is shown in Figure 1.

### 3.1 Single-cell transcriptomic analysis reveals SCLC subtype heterogeneity and CMA activity distribution

**FIGURE 2:**
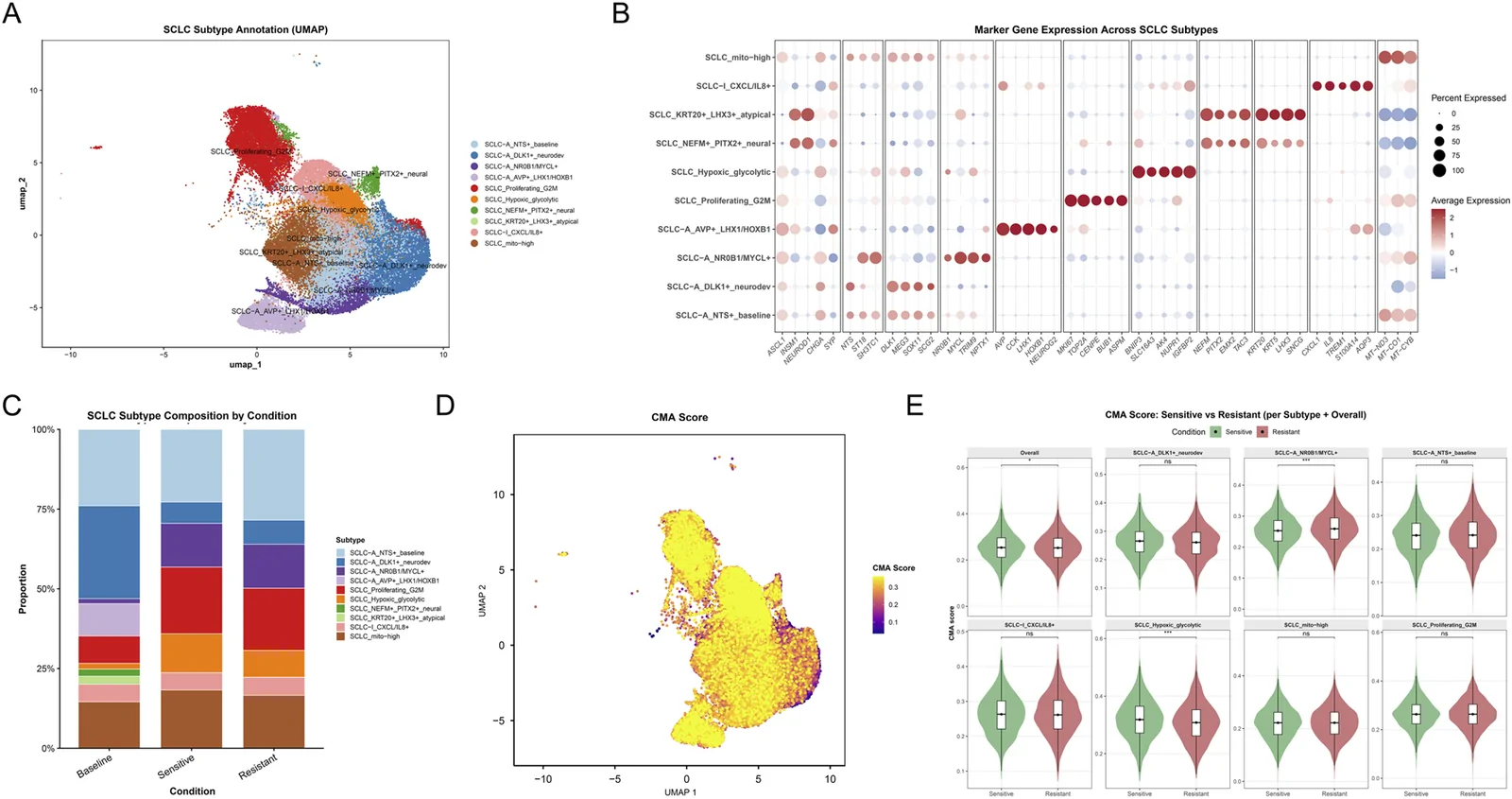
Single-cell characterization of SCLC subtypes and CMA activity across treatment-response states. **(A)** UMAP visualization of SCLC cells annotated into distinct molecular subtypes. **(B)** Dot plot showing the expression patterns of representative marker genes across annotated SCLC subtypes. **(C)** Stacked bar plot showing the relative proportions of SCLC subtypes across baseline, sensitive, and resistant conditions. **(D)** UMAP visualization of single-cell CMA scores across SCLC cells. **(E)** Violin plots comparing CMA scores between sensitive and resistant cells in the overall SCLC population and within each annotated subtype. ns, not significant; *P < 0.05; **P < 0.01; ***P < 0.001; ****P < 0.0001.

To systematically characterize CMA-related heterogeneity in SCLC, we first performed dimensionality reduction, clustering, and subtype annotation of SCLC cells based on scRNA-seq data. UMAP visualization showed that SCLC cells were not uniformly distributed but instead formed multiple cell clusters with distinct molecular features, indicating substantial transcriptomic heterogeneity within SCLC (Figure 2A). Based on canonical SCLC subtype markers and functionally relevant gene expression patterns, the cells were further annotated as SCLC-A_NTS_baseline, SCLC-A_DLK1_neurodev, SCLC-A_NR0B1/MYCL+, SCLC-A_AVP_LHX1/HOXB1, SCLC_Proliferating_G2M, SCLC_Hypoxia_glycolytic, SCLC_NEFM_PITX2_neural, SCLC_KRT20_LHX3_atypical, SCLC_CXCL8+, and SCLC_mito-high subtypes. The marker gene dot plot further showed distinct expression patterns related to neuroendocrine features, proliferative status, hypoxia-glycolytic programs, inflammatory chemokine expression, and mitochondrial characteristics across different subtypes, supporting the reliability of the subtype annotation (Figure 2B).

We next compared the composition of SCLC subtypes across baseline, sensitive, and resistant states. The results showed evident differences in subtype proportions among these states, suggesting that treatment-response status may be accompanied by remodeling of the SCLC cellular composition (Figure 2C). Specifically, the proportion of SCLC-A_DLK1_neurodev cells was higher in the baseline group than in the sensitive and resistant groups, whereas SCLC-A_NR0B1/MYCL+ and SCLC_Hypoxia_glycolytic cells accounted for relatively lower proportions in the baseline group. In addition, several subtypes exhibited distinct distribution patterns between the sensitive and resistant groups, indicating that differences in SCLC treatment sensitivity may be related not only to isolated molecular alterations but also to dynamic changes in tumor cell subtype composition.

Furthermore, we calculated single-cell CMA scores based on the CMA-related gene set and visualized their distribution in the UMAP space. CMA activity showed a heterogeneous distribution among SCLC cells, with apparent differences in CMA scores across distinct cellular regions, suggesting that CMA was not uniformly activated across all SCLC cells but instead displayed subtype-associated heterogeneity (Figure 2D). At the overall cell population level, CMA scores differed significantly between resistant and sensitive cells, suggesting a potential association between CMA activity and treatment resistance (Figure 2E). Further stratified analysis across SCLC subtypes showed that significant differences in CMA scores between sensitive and resistant cells were observed in the SCLC-A_NR0B1/MYCL+ and SCLC_Hypoxia_glycolytic subtypes, whereas other subtypes did not show significant differences. These findings indicate that CMA-related alterations may depend on specific SCLC molecular subtype contexts (Figure 2E).

### 3.2 WGCNA identifies key gene modules associated with CMA activity

**FIGURE 3:**
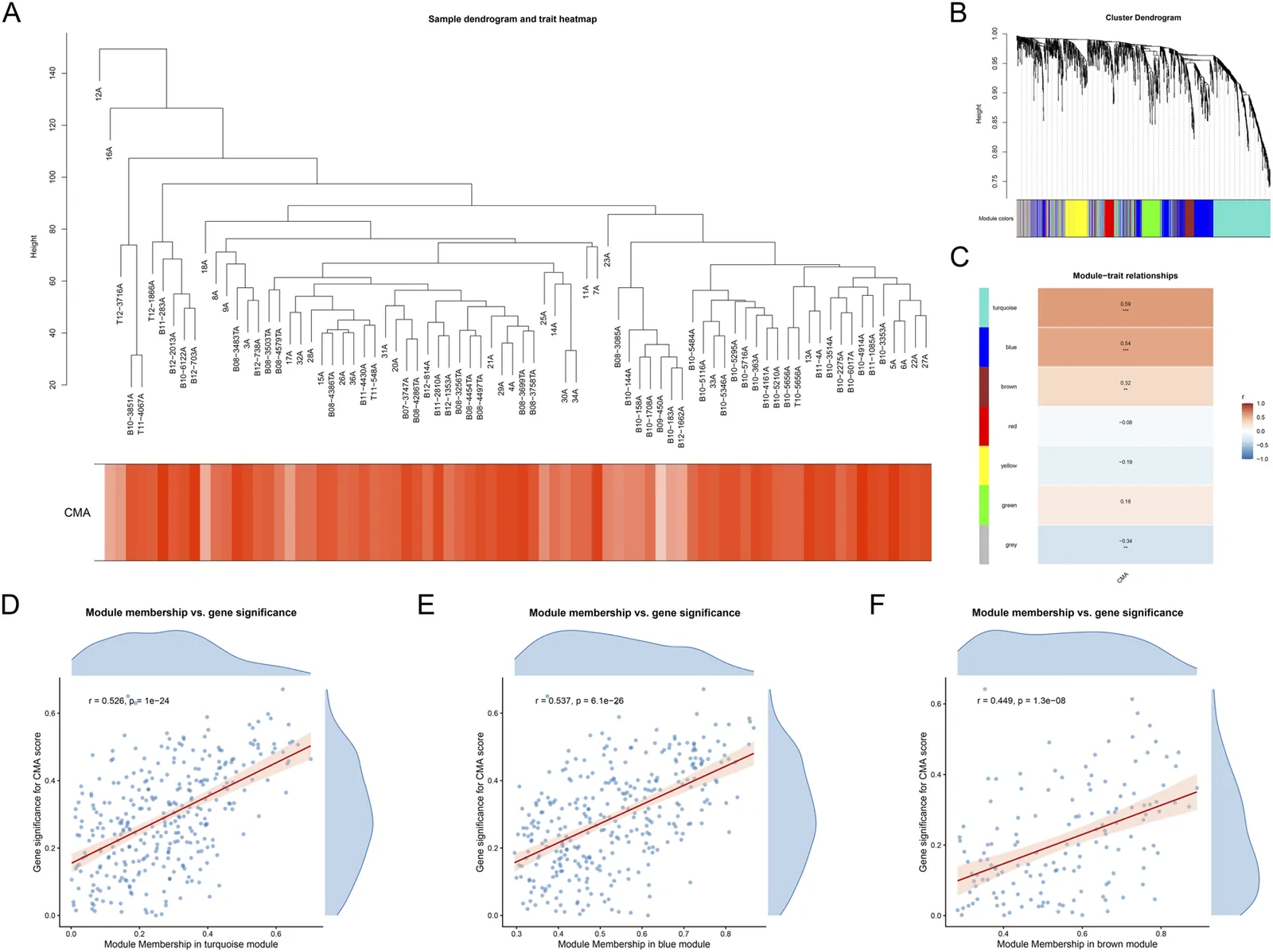
Identification of CMA-related gene modules by WGCNA. **(A)** Sample dendrogram and trait heatmap showing hierarchical clustering of samples and the distribution of CMA scores. **(B)** Gene cluster dendrogram displaying co-expression modules identified by WGCNA, with different colors representing distinct modules. **(C)** Heatmap showing correlations between module eigengenes and CMA scores. **(D)** Scatter plot showing the relationship between module membership and gene significance for CMA scores in the turquoise module. **(E)** Scatter plot showing the relationship between module membership and gene significance for CMA scores in the blue module. **(F)** Scatter plot showing the relationship between module membership and gene significance for CMA scores in the brown module. ns, not significant; *P < 0.05; **P < 0.01; ***P < 0.001; ****P < 0.0001.

After single-cell analysis suggested subtype-dependent and resistance-associated differences in CMA activity, WGCNA was further performed at the bulk transcriptomic level to identify gene modules associated with the CMA score. First, a sample clustering dendrogram was constructed based on the expression profiles and combined with the CMA score to generate a sample-trait heatmap. The results showed certain differences in overall expression patterns among samples, but no obvious outlier samples were observed, indicating that the included samples were suitable for subsequent co-expression network construction (Figure 3A).

Subsequently, a co-expression network was constructed based on gene expression correlations, and hierarchical clustering was used to identify distinct gene modules. The module clustering dendrogram showed that genes were divided into multiple color-labeled co-expression modules, suggesting the presence of gene sets with similar expression patterns in the bulk transcriptomic data (Figure 3B). Correlation analysis between module eigengenes and the CMA score revealed clear differences in both the direction and strength of associations across modules. Among them, the turquoise module showed the strongest positive correlation with the CMA score (r = 0.59, P < 0.001), followed by the blue module (r = 0.54, P < 0.001) and the brown module (r = 0.32, P < 0.001). In contrast, the grey module was negatively correlated with the CMA score (r = −0.34, P < 0.001), whereas the red, yellow, and green modules showed relatively weaker correlations (Figure 3C).

To further evaluate the robustness of the association between genes within key modules and CMA activity, the relationships between module membership and gene significance for the CMA score were analyzed in the turquoise, blue, and brown modules. A significant positive correlation was observed between module membership and gene significance in the turquoise module (r = 0.526, P < 0.0001) (Figure 3D). Similar positive correlations were also observed in the blue module (r = 0.537, P < 0.0001) (Figure 3E) and the brown module (r = 0.449, P < 0.0001) (Figure 3F).

WGCNA identified the turquoise, blue, and brown modules as key modules positively associated with the CMA score. Although the grey module was negatively correlated with the CMA score, it was not included because the downstream screening focused on genes positively associated with CMA activity. Therefore, genes from the turquoise, blue, and brown modules were merged as the CMA-related module gene set for subsequent candidate gene screening.

### 3.3 Differential expression analysis and WGCNA-based intersection screening identify CMA-related candidate genes

**FIGURE 4:**
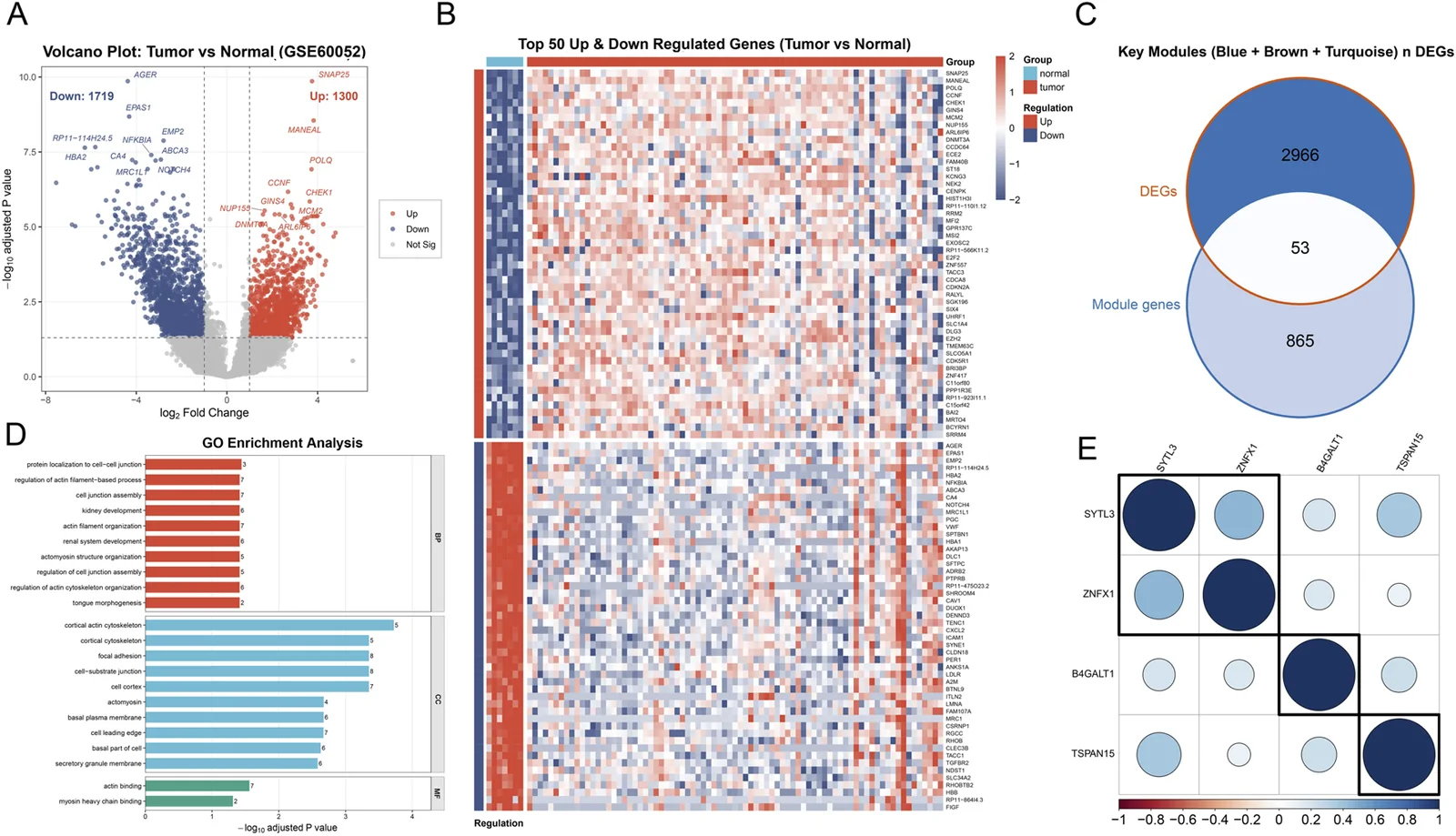
Identification and functional characterization of CMA-related candidate genes. **(A)** Volcano plot showing differentially expressed genes between SCLC tumor and normal samples in the GSE60052 cohort. Red and blue dots indicate upregulated and downregulated genes, respectively. **(B)** Heatmap displaying the expression patterns of representative upregulated and downregulated genes between tumor and normal samples. **(C)** Venn diagram showing the intersection between differentially expressed genes and CMA-related module genes identified by WGCNA, resulting in 53 overlapping candidate genes. **(D)** GO enrichment analysis of the 53 candidate genes, including biological process (BP), cellular component (CC), and molecular function (MF) categories. **(E)** Correlation analysis of key genes retained for subsequent model construction.

Following the single-cell characterization of CMA-related heterogeneity across SCLC subtypes and treatment-response states, we next performed bulk-level candidate gene screening. After identifying WGCNA key modules significantly associated with the CMA score, tumor versus normal differential expression analysis was integrated to screen CMA-related candidate genes. First, gene expression differences between SCLC tumor and normal tissues were analyzed using the GSE60052 cohort. The volcano plot revealed marked transcriptomic differences between tumor and normal samples, with 1,300 upregulated genes and 1,719 downregulated genes identified (Figure 4A). Among them, SNAP25, MANEAL, POLQ, CCNF, and CHEK1 were upregulated in tumor tissues, whereas AGER, EPAS1, EMP2, ABCA3, and CA4 were downregulated. The heatmap of differentially expressed genes further displayed the expression patterns of top-ranked upregulated and downregulated genes. Tumor and normal samples were clearly separated based on these expression profiles, suggesting that these genes reflected the molecular alterations of SCLC tumor tissues relative to normal tissues (Figure 4B).

Next, differentially expressed genes from the GSE60052 tumor versus normal analysis were intersected with CMA-related key module genes identified by WGCNA. Because the turquoise, blue, and brown modules were all significantly positively correlated with the CMA score, genes from these three modules were combined as the CMA-related module gene set. The Venn diagram showed 53 overlapping genes between differentially expressed genes and key module genes (Figure 4C). These genes exhibited both tumor-associated differential expression and bulk-level CMA-related co-expression characteristics, and were therefore defined as CMA-related candidate genes for subsequent model construction.

To further explore the potential biological functions of these candidate genes, GO enrichment analysis was performed for the 53 overlapping genes. BP analysis showed that these genes were mainly enriched in protein localization to cell-cell junction, regulation of actin filament-based process, and cell junction assembly, suggesting their potential involvement in cell-cell junction organization and cytoskeletal regulation (Figure 4D). CC analysis indicated that these genes were primarily localized to the cortical actin cytoskeleton, cortical cytoskeleton, and focal adhesion. MF analysis further suggested that the candidate genes were mainly associated with actin binding and myosin heavy chain binding (Figure 4D). These findings indicate that the candidate genes may be closely related to cytoskeletal remodeling, cell junction organization, cell adhesion, and cell motility.

Furthermore, correlation analysis was performed for key genes retained in the subsequent model. The results showed a relatively clear positive correlation between SYTL3 and ZNFX1, whereas correlations among other gene pairs were weaker (Figure 4E). This finding suggests that the model genes were not highly redundant and may provide complementary information for subsequent multi-gene prognostic model construction.

### 3.4 Machine learning-based selection of core genes and construction of the CMA-related prognostic model

**FIGURE 5:**
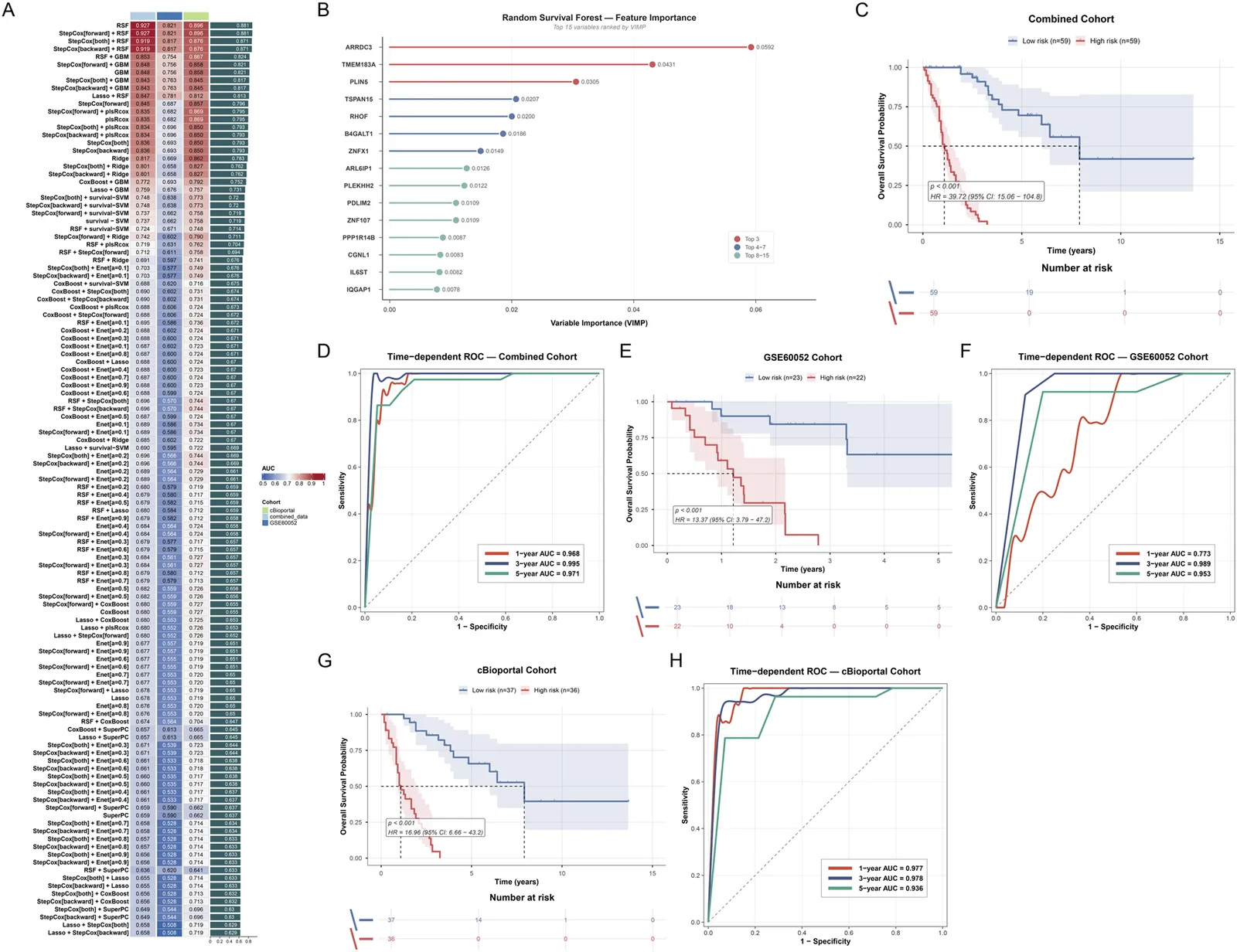
Construction and evaluation of the CMA-related prognostic model using machine learning. **(A)** C-index comparison of 101 machine learning algorithm combinations across the combined cohort, GSE60052 cohort, and cBioPortal cohort. **(B)** Variable importance ranking of candidate genes in the RSF model. **(C)** Kaplan-Meier survival analysis of high-risk and low-risk groups in the combined cohort. **(D)** Time-dependent ROC curves evaluating the 1-, 3-, and 5-year predictive performance of the Risk Score in the combined cohort. **(E)** Kaplan-Meier survival analysis of high-risk and low-risk groups in the GSE60052 cohort. **(F)** Time-dependent ROC curves for 1-, 3-, and 5-year survival prediction in the GSE60052 cohort. **(G)** Kaplan-Meier survival analysis of high-risk and low-risk groups in the cBioPortal cohort. **(H)** Time-dependent ROC curves for 1-, 3-, and 5-year survival prediction in the cBioPortal cohort. P values in Kaplan-Meier analyses were calculated using the log-rank test.

After obtaining 53 CMA-related candidate genes, we further applied multiple machine learning algorithm combinations to construct a prognostic model and compared their predictive performance across different cohorts. Based on the candidate gene expression matrix and survival information, the C-index performance of 101 machine learning algorithm combinations was systematically evaluated. The results showed marked differences in predictive ability among different algorithm combinations. Among them, StepCox [forward] + RSF and RSF both showed high and stable C-index performance across the combined cohort, GSE60052 cohort, and cBioPortal cohort (Figure 5A). Although StepCox [forward] + RSF also achieved favorable performance, the standalone RSF model maintained comparable cross-cohort predictive performance while avoiding an additional stepwise feature-selection procedure. Therefore, RSF was selected as the final modeling method because it provided a balance between predictive performance, model simplicity, and reduced risk of overfitting.

Candidate gene importance was then evaluated based on the RSF model. Variable importance analysis showed that ARRDC3, TMEM183A, and PLIN5 ranked as the top three genes, suggesting that they contributed substantially to prognostic prediction in the model (Figure 5B). The model was then used to calculate the Risk Score for each patient, which was used for subsequent risk stratification and prognostic evaluation.

Patients were stratified into high-risk and low-risk groups according to the median Risk Score. In the combined cohort, Kaplan-Meier survival analysis showed that patients in the high-risk group had significantly shorter OS than those in the low-risk group, indicating that the Risk Score effectively distinguished SCLC patients with different prognostic outcomes (Figure 5C). Time-dependent ROC curves further demonstrated that the Risk Score had favorable predictive performance for 1-, 3-, and 5-year OS in the combined cohort (Figure 5D).

The model performance was further evaluated in the GSE60052 and cBioPortal cohorts. In the GSE60052 cohort, patients in the high-risk group also exhibited poorer OS, with a clear separation between the survival curves of the two groups (Figure 5E). Corresponding time-dependent ROC analysis showed that the Risk Score had favorable predictive efficiency for 1-, 3-, and 5-year OS (Figure 5F). In the cBioPortal cohort, high-risk patients continued to show significantly worse survival outcomes than low-risk patients, indicating that the model had certain stability across different data sources (Figure 5G). ROC analysis further supported the prognostic predictive value of the Risk Score in the cBioPortal cohort (Figure 5H). Together, these results indicate that the RSF-based CMA-related Risk Score can robustly stratify survival risk in patients with SCLC across multiple cohorts.

### 3.5 Clinical associations and subgroup prognostic evaluation of the risk score

**FIGURE 6:**
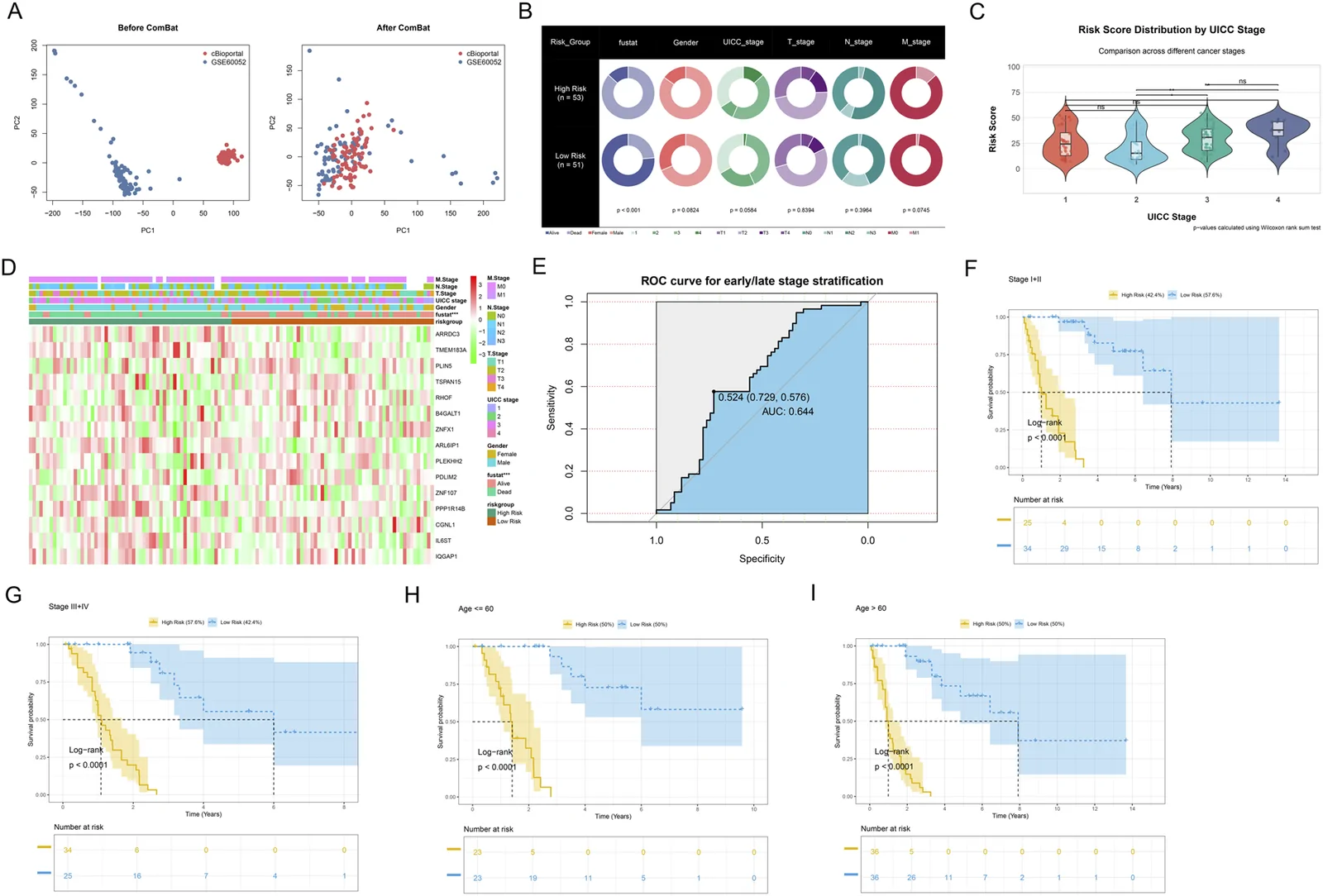
Clinical association and subgroup prognostic evaluation of the CMA-related Risk Score. **(A)** Principal component analysis showing the distribution of GSE60052 and cBioPortal samples before and after batch-effect correction. **(B)** Circular bar plots showing the associations between risk groups and clinical characteristics, including survival status, sex, UICC stage, T stage, N stage, and M stage. **(C)** Comparison of Risk Score distributions among different UICC stages. **(D)** Heatmap showing the expression patterns of model genes and their relationships with risk group and clinical annotations. **(E)** ROC curve exploring the association between the Risk Score and UICC stage status. **(F)** Kaplan-Meier survival analysis of high-risk and low-risk groups in patients with UICC stage I-II disease. **(G)** Kaplan-Meier survival analysis of high-risk and low-risk groups in patients with UICC stage III-IV disease. **(H)** Kaplan-Meier survival analysis of high-risk and low-risk groups in patients aged ≤60 years. **(I)** Kaplan-Meier survival analysis of high-risk and low-risk groups in patients aged >60 years. ns, not significant; *P < 0.05; **P < 0.01; ***P < 0.001; ****P < 0.0001.

To integrate the GSE60052 and cBioPortal cohorts, batch-effect correction was first performed for the two bulk transcriptomic datasets. PCA showed that samples from the two cohorts were clearly separated in the expression space before correction, indicating evident batch differences. After ComBat correction, samples from the two cohorts became more intermixed, suggesting that batch effects were effectively reduced and providing a basis for subsequent analyses in the combined cohort (Figure 6A).

We next analyzed the associations between Risk Score-based grouping and clinicopathological characteristics. Circular bar plots showed a significant difference in survival status between high-risk and low-risk groups, with a higher proportion of death events observed in the high-risk group (P < 0.001). In contrast, risk group was not significantly associated with sex, UICC stage, T stage, N stage, or M stage, suggesting that the prognostic differences reflected by the Risk Score were not entirely dependent on conventional clinical stage or sex-related factors (Figure 6B).

Further comparison of Risk Score distributions across different UICC stages showed that the Risk Score differed to some extent among disease stages (Figure 6C). Specifically, significant differences were observed between stage II and stage III, between stage II and stage IV, and between stage I and stage IV. Overall, the Risk Score tended to increase with UICC stage progression, although this trend did not reach statistical significance across all pairwise stage comparisons.

A heatmap was then generated to visualize the expression patterns of model genes and their relationships with clinical characteristics. The results showed that model-related genes exhibited heterogeneous expression patterns across samples, with differences in gene expression observed between high-risk and low-risk groups. Combined with the clinical annotations shown above the heatmap, risk group showed a clear relationship with survival status, whereas its correspondence with UICC stage, T stage, N stage, and M stage was relatively limited. These findings suggest that the Risk Score captures molecular risk features distinct from conventional staging systems (Figure 6D).

To further explore whether the Risk Score was associated with disease stage status, patients were classified into UICC stage I-II and UICC stage III-IV groups, and ROC analysis was performed. The Risk Score showed modest discriminatory performance between UICC stage I-II and UICC stage III-IV disease, with an AUC of 0.644, suggesting that the Risk Score was related to disease progression status, although its staging-discrimination ability was limited (Figure 6E).

Finally, we evaluated the prognostic stratification ability of the Risk Score across different clinical subgroups. Among patients with UICC stage I-II disease, the high-risk group had significantly shorter OS than the low-risk group, indicating that the Risk Score could identify patients with poor prognosis even in early-stage disease (Figure 6F). Among patients with UICC stage III-IV disease, the high-risk group also showed markedly worse survival outcomes, suggesting that the Risk Score retained prognostic discrimination in advanced-stage disease (Figure 6G). After stratification by age, high-risk patients consistently exhibited significantly poorer OS in both the ≤60-year and >60-year subgroups (Figures 6H,I). These results indicate that the CMA-related Risk Score maintained stable prognostic value across different clinical subgroups.

### 3.6 Independent prognostic value and functional pathway characteristics of the risk score

**FIGURE 7:**
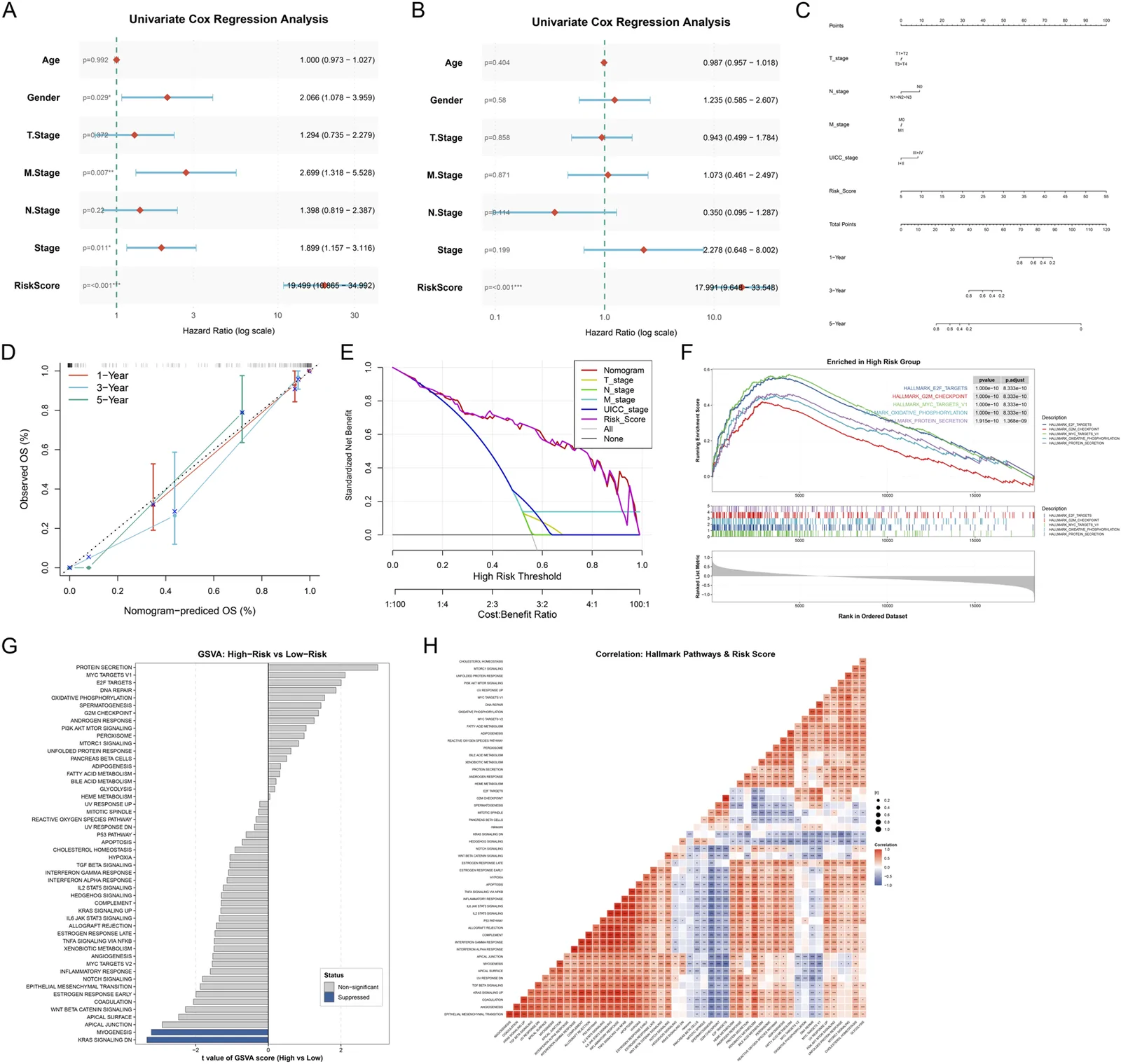
Independent prognostic value and functional pathway characteristics of the CMA-related Risk Score. **(A)** Univariate Cox regression analysis of the Risk Score and clinicopathological variables for overall survival. **(B)** Multivariate Cox regression analysis evaluating the independent prognostic value of the Risk Score. **(C)** Nomogram integrating the Risk Score and clinical variables to predict 1-, 3-, and 5-year overall survival. **(D)** Calibration curves assessing the agreement between nomogram-predicted and observed overall survival probabilities. **(E)** Decision curve analysis evaluating the clinical net benefit of the nomogram. **(F)** GSEA showing Hallmark pathways significantly enriched in the high-risk group, including MYC targets V1, E2F targets, protein secretion, oxidative phosphorylation, and G2M checkpoint. **(G)** GSVA-based comparison of Hallmark pathway activities between high-risk and low-risk groups, showing suppressed KRAS signaling up and WNT beta-catenin signaling in the high-risk group. **(H)** Correlation heatmap showing associations between the Risk Score and Hallmark pathway activities. ns, not significant; *P < 0.05; **P < 0.01; ***P < 0.001; ****P < 0.0001.

To further evaluate whether the CMA-related Risk Score had independent prognostic value, univariate Cox regression analysis was first performed in the combined cohort. The results showed that the Risk Score was significantly associated with OS, with higher risk scores indicating poorer survival outcomes (Figure 7A). After incorporating the Risk Score and clinicopathological variables into multivariate Cox regression analysis, the Risk Score retained a significant prognostic association, suggesting that it may serve as an independent prognostic factor for patients with SCLC (Figure 7B).

Based on these findings, a nomogram integrating the Risk Score and clinical variables was constructed to predict 1-, 3-, and 5-year OS probabilities. The nomogram showed that the Risk Score contributed substantially to the total point system, indicating its important role in individualized survival prediction (Figure 7C). Calibration curves further demonstrated good agreement between the predicted and observed 1-, 3-, and 5-year survival probabilities, suggesting favorable calibration performance of the model (Figure 7D). Decision curve analysis showed that the nomogram provided a higher net benefit than the “treat-all” or “treat-none” strategies across a range of threshold probabilities, indicating its potential clinical utility (Figure 7E).

We next compared functional pathway differences between high-risk and low-risk groups. GSEA showed that five Hallmark pathways, including MYC targets V1, E2F targets, protein secretion, oxidative phosphorylation, and G2M checkpoint, were significantly enriched in the high-risk group (P < 0.001, adjusted P < 0.001) (Figure 7F). Among them, the enrichment curves of MYC targets V1 and E2F targets showed prominent peaks, suggesting that the high-risk group was closely associated with proliferation-related transcriptional programs. In addition, enrichment of G2M checkpoint, protein secretion, and oxidative phosphorylation indicated that the high-risk state may be accompanied by enhanced cell-cycle progression, secretory activity, and energy metabolism.

GSVA was further used to evaluate differences in Hallmark pathway activity between high-risk and low-risk groups. Among the displayed Hallmark pathways, most did not show statistically significant differences between the two groups. Only KRAS signaling up and WNT beta-catenin signaling were suppressed in the high-risk group, suggesting that the significant GSVA differences between risk groups were mainly concentrated in these two signaling pathways (Figure 7G).

Finally, we analyzed the correlations between the Risk Score and Hallmark pathway activities. The results showed that the Risk Score was correlated with multiple Hallmark pathways, with some proliferation-, cell cycle-, and metabolism-related pathways showing overall positive correlation trends, whereas several other pathways showed negative correlation trends (Figure 7H). These findings indicate that the CMA-related Risk Score not only has prognostic predictive value but also reflects distinct functional pathway states in patients with SCLC.

### 3.7 Immune microenvironment characteristics associated with the risk score

**FIGURE 8:**
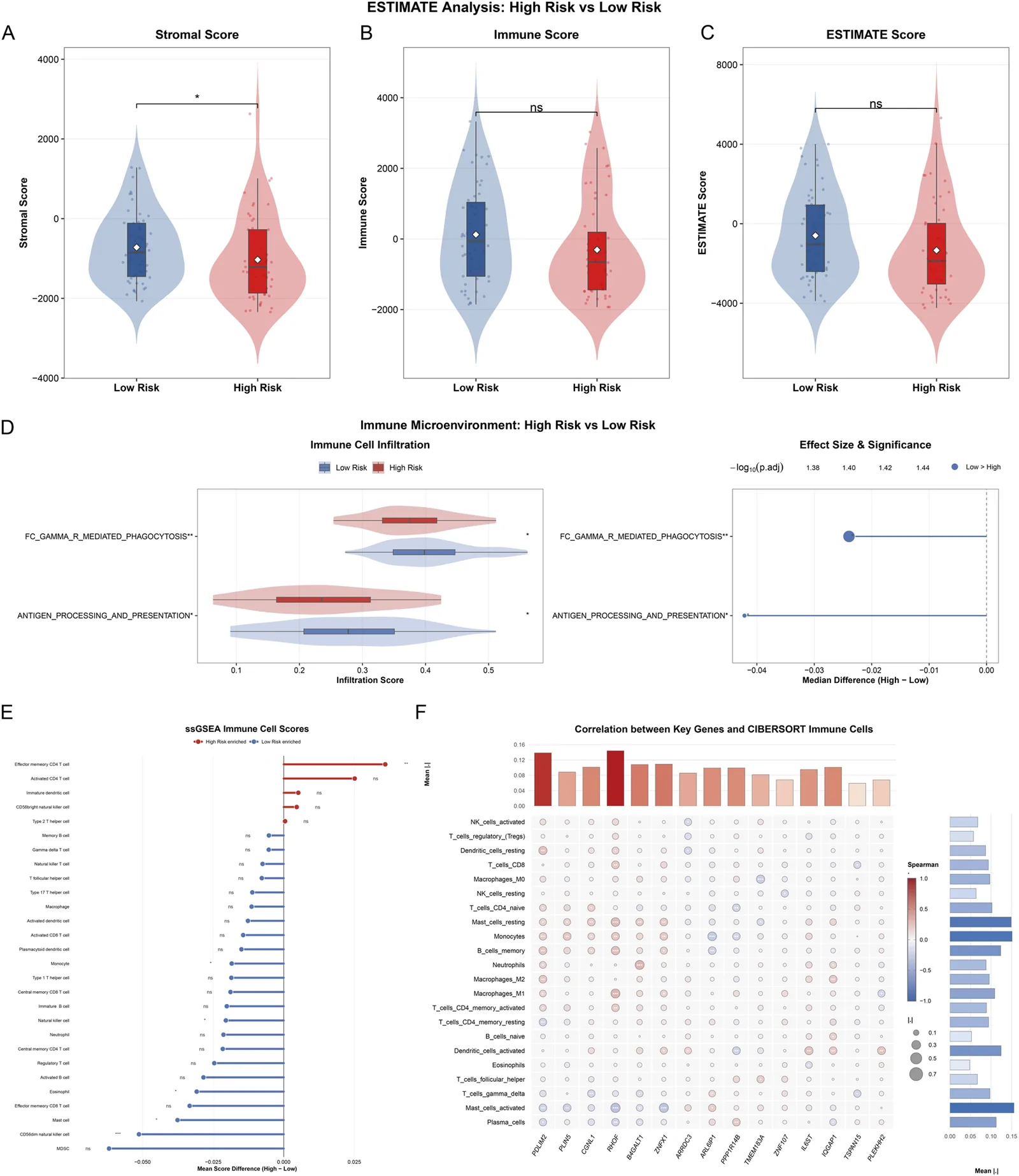
Immune microenvironment characteristics associated with the CMA-related Risk Score. **(A)** Comparison of Stromal Score between high-risk and low-risk groups based on the ESTIMATE algorithm. **(B)** Comparison of Immune Score between high-risk and low-risk groups. **(C)** Comparison of ESTIMATE Score between high-risk and low-risk groups. **(D)** Differences in immune-related functional pathways between high-risk and low-risk groups. **(E)** ssGSEA-based comparison of immune cell infiltration between high-risk and low-risk groups. **(F)** Correlation heatmap showing associations between model genes and CIBERSORT-inferred immune cell populations. ns, not significant; *P < 0.05; **P < 0.01; ***P < 0.001; ****P < 0.0001.

To further evaluate whether the CMA-related Risk Score was associated with the tumor immune microenvironment, we first applied the ESTIMATE algorithm to compare stromal and immune components between high-risk and low-risk groups. The results showed that the Stromal Score was significantly lower in the high-risk group than in the low-risk group, suggesting a relative reduction in stromal components in high-risk tumors (Figure 8A). In contrast, no statistically significant differences were observed in the Immune Score or ESTIMATE Score between the two groups, indicating that overall immune infiltration and the integrated microenvironment score were not markedly different (Figures 8B,C).

We next analyzed differences in immune function-related pathway activities. The results showed significant differences in Fc gamma receptor-mediated phagocytosis and antigen processing and presentation between high-risk and low-risk groups, with effect size analysis indicating higher activity of these functions in the low-risk group (Figure 8D). These findings suggest that low-risk patients may have relatively more active antigen processing, antigen presentation, and phagocytosis-related immune functional states.

To further characterize differences in specific immune cell populations, ssGSEA was used to estimate the relative enrichment of immune cell types between the two risk groups. The results showed that several T cell-related subsets were relatively increased in the high-risk group, with effector memory CD4 T cells showing a relatively evident difference. In contrast, CD56dim natural killer cells and several other immune cell subsets were significantly decreased in the high-risk group (Figure 8E). Overall, immune cell differences between risk groups did not reflect a unidirectional increase or decrease in global immune activity, but rather a redistribution of specific immune cell subsets.

Finally, we analyzed the correlations between key model genes and CIBERSORT-inferred immune cell proportions. The results revealed heterogeneous correlation patterns between different model genes and multiple immune cell subsets, suggesting that CMA-related model genes may be associated with the composition of the immune microenvironment (Figure 8F). In particular, some genes showed varying degrees of positive or negative correlations with monocytes, macrophages, dendritic cells, mast cells, and T cell-related subsets. Taken together, these findings suggest that high-risk and low-risk groups differ mainly in stromal components, selected immune functional pathways, and specific immune cell subsets. Although Immune Score and ESTIMATE Score were not significantly different between the two groups, the observed differences in Stromal Score, immune functional pathways, and specific immune cell populations indicate that the Risk Score is associated with selected immune microenvironment features.

### 3.8 Drug sensitivity differences associated with the risk score and functional validation of ARRDC3

**FIGURE 9:**
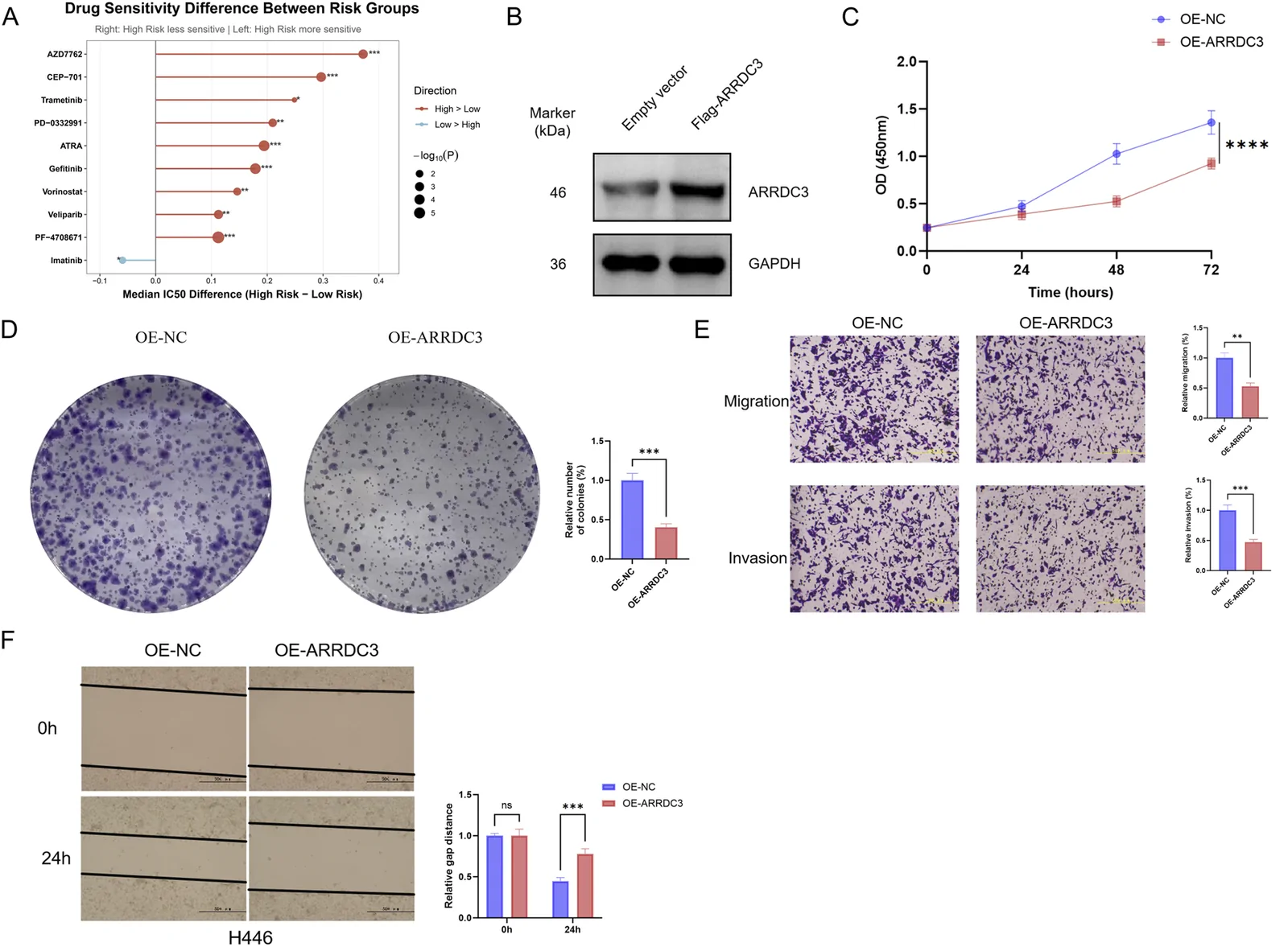
Drug sensitivity differences and functional validation of ARRDC3. **(A)** Predicted drug sensitivity differences between high-risk and low-risk groups for 10 representative therapeutic agents. The x-axis represents the median IC50 difference between high-risk and low-risk groups; positive values indicate higher predicted IC50 in the high-risk group, whereas negative values indicate lower predicted IC50 in the high-risk group. Dot size indicates −log10(P). **(B)** Western blot analysis confirming ARRDC3 overexpression in H446 cells. **(C)** CCK-8 assay showing the effect of ARRDC3 overexpression on H446 cell proliferation. **(D)** Colony formation assay evaluating the effect of ARRDC3 overexpression on clonogenic capacity. **(E)** Transwell migration and invasion assays assessing the effect of ARRDC3 overexpression on cell motility and invasiveness. **(F)** Wound-healing assay evaluating the effect of ARRDC3 overexpression on cell migration. ns, not significant; *P < 0.05; **P < 0.01; ***P < 0.001; ****P < 0.0001.

To further evaluate the potential relevance of the CMA-related Risk Score in therapeutic response stratification, we compared predicted drug sensitivity to 10 representative agents between high-risk and low-risk groups. The results showed that, except for Imatinib, the other nine drugs exhibited higher predicted IC50 values in the high-risk group, suggesting that high-risk patients may have lower sensitivity to AZD7762, CEP-701, Trametinib, PD-0332991, ATRA, Gefitinib, Vorinostat, Veliparib, and PF-4708671. In contrast, Imatinib showed a lower predicted IC50 in the high-risk group, indicating that high-risk patients may have relatively higher potential sensitivity to this agent (Figure 9A). These drugs cover multiple therapeutic directions, including DNA damage response, MAPK/ERK signaling, cell-cycle regulation, receptor tyrosine kinase signaling, epigenetic regulation, PI3K/AKT/mTOR signaling, and differentiation induction, suggesting that CMA-related risk stratification may be associated with distinct drug response patterns.

Given that ARRDC3 showed high variable importance in the RSF model, we further performed *in vitro* functional validation in H446 cells. Western blot analysis showed that ARRDC3 protein expression was markedly increased in the ARRDC3 overexpression group compared with the empty vector control group, confirming the successful establishment of the ARRDC3 overexpression system (Figure 9B). CCK-8 assays subsequently showed that ARRDC3 overexpression significantly reduced the proliferative capacity of H446 cells, suggesting that ARRDC3 suppresses SCLC cell proliferation (Figure 9C). Colony formation assays further showed that the number of colonies was markedly decreased in the ARRDC3 overexpression group, indicating that ARRDC3 inhibits long-term proliferative and clonogenic capacities (Figure 9D).

Further migration and invasion assays showed that ARRDC3 overexpression significantly reduced the migratory and invasive abilities of H446 cells (Figure 9E). Consistently, wound healing assays showed that the ARRDC3 overexpression group had a larger relative wound gap after 24 h, suggesting impaired cell migration (Figure 9F). Overall, these *in vitro* results demonstrate that ARRDC3 overexpression suppresses proliferation, colony formation, migration, and invasion in H446 cells, supporting ARRDC3 as a functionally relevant protective gene rather than merely a model-associated marker.

## 4 Discussion

This study integrated single-cell transcriptomics, bulk transcriptomics, machine learning modeling, and *in vitro* experimental validation to systematically evaluate CMA-related heterogeneity and its clinical significance in SCLC. Unlike conventional analyses that treat CMA as a global molecular event, our findings suggest that CMA activity is not uniformly distributed across all SCLC cells but is closely associated with specific cellular subtypes and treatment-response states. The CMA-related Risk Score established in this study robustly stratified patients into high-risk and low-risk groups across multiple cohorts and reflected multi-layered biological features, including proliferation, metabolism, immune microenvironment characteristics, and potential drug response patterns.

The highly aggressive nature of SCLC is closely related to its marked molecular heterogeneity. Baine et al. (2020) systematically characterized SCLC subtypes defined by ASCL1, NEUROD1, POU2F3, and YAP1, whereas Gay et al. (2021) further proposed four major SCLC subtypes with distinct transcription factor programs, immune pathway activation, and therapeutic vulnerabilities. Megyesfalvi et al. (2022) and Qi et al. (2022) further supported the clinical and prognostic relevance of subtype-specific transcription factor expression in independent SCLC cohorts. At the single-cell level, Chan et al. (2021) and Tian et al. (2022) demonstrated pronounced cellular heterogeneity, plasticity, and microenvironmental diversity in SCLC. Our single-cell findings further support this concept. SCLC cells formed multiple transcriptionally distinct subpopulations in the UMAP space, and subtype proportions differed across baseline, sensitive, and resistant states. Specifically, SCLC-A_DLK1_neurodev cells accounted for a higher proportion in the baseline group, whereas SCLC-A_NR0B1/MYCL+ and SCLC_Hypoxia_glycolytic cells were relatively less abundant in the baseline group, suggesting that treatment may be accompanied by remodeling of specific cellular states. Notably, CMA scores showed evident heterogeneity across SCLC cells, and significant differences between sensitive and resistant cells were observed in the SCLC-A_NR0B1/MYCL+ and SCLC_Hypoxia_glycolytic subtypes. Given the role of CMA in proteostasis maintenance, metabolic regulation, and stress adaptation, altered CMA activity in resistant cells may reflect adaptive remodeling of tumor cells under therapeutic pressure. For a rapidly proliferating and treatment-resistant malignancy such as SCLC, CMA-related cellular states may provide a new entry point for understanding resistance evolution under therapeutic stress.

The model construction strategy used in this study differs from conventional bulk transcriptome-based prognostic models. Although many reported prognostic signatures are generated directly from bulk transcriptomic data, such strategies may be affected by mixed cellular composition and cohort heterogeneity. Kuksin et al. (2021) emphasized that bulk RNA-seq provides valuable cohort-level transcriptomic and clinical information but cannot fully resolve cell-type-specific or cell-state-specific contributions, whereas single-cell sequencing enables cellular-resolution characterization of tumor heterogeneity. Consistent with this rationale, our study first identified CMA heterogeneity and treatment response-associated cellular states at the single-cell level, followed by integration with WGCNA, tumor versus normal differential expression analysis, and machine learning modeling. Therefore, the resulting Risk Score is not merely a statistically selected survival-associated gene set but more closely resembles a molecular risk model derived from specific cellular-state programs. Functional analyses further supported this interpretation. The high-risk group was significantly enriched in MYC targets V1, E2F targets, G2M checkpoint, oxidative phosphorylation, and protein secretion pathways. George et al. (2015) reported that TP53 and RB1 are nearly universally inactivated in SCLC, establishing cell-cycle deregulation as a central biological feature of this disease. Mollaoglu et al. (2017) further showed that MYC cooperates with Rb1 and Trp53 loss to promote aggressive and highly metastatic SCLC and drives a variant neuroendocrine subtype with specific therapeutic vulnerabilities. Ireland et al. (2020) demonstrated that MYC can reprogram neuroendocrine fate and drive temporal evolution of SCLC subtypes, linking MYC activity to cellular-state plasticity. Therefore, enrichment of MYC targets V1, E2F targets, and G2M checkpoint in the high-risk group is consistent with a proliferative and cell-cycle-active malignant state captured by the Risk Score. In addition, oxidative phosphorylation enrichment may suggest that high-risk tumors exhibit metabolic adaptation beyond glycolysis alone. Ashton et al. (2018) and Uslu et al. (2024) highlighted the role of oxidative phosphorylation in cancer metabolic adaptation, therapeutic resistance, and metastatic potential. Together, these transcriptomic associations suggest that a higher Risk Score may reflect a malignant cellular state related to proliferation, cell-cycle progression, and metabolic adaptation, although direct mechanistic validation is still required.

Immune microenvironment and drug sensitivity analyses further expanded the biological interpretation of the Risk Score. SCLC is generally considered to exhibit a relatively immune-cold microenvironment. Chen Y. et al. (2023) and Caliman et al. (2023) reported that SCLC is characterized by limited immune infiltration, impaired antigen presentation, and downregulation of major histocompatibility complex molecules, which may contribute to insufficient antitumor immune activation. Consistent with these observations, the overall Immune Score and ESTIMATE Score did not differ significantly between high-risk and low-risk groups, whereas differences were mainly observed in Stromal Score, antigen processing and presentation, and selected immune cell subsets. These findings suggest that the immune relevance of the Risk Score may be reflected more by specific stromal and immune features than by global immune infiltration levels. Drug sensitivity prediction showed that the high-risk group had higher predicted IC50 values for most representative agents, involving DNA damage response, MAPK/ERK signaling, receptor tyrosine kinase signaling, cell-cycle regulation, and epigenetic regulation. Sen et al. highlighted DNA damage repair as an important therapeutic vulnerability in SCLC, suggesting that the predicted drug sensitivity patterns observed in the high-risk group may reflect broader therapeutic adaptation rather than a single molecular dependency (Sen et al., 2018). However, these findings should be interpreted as exploratory and hypothesis-generating computational predictions. Further pharmacological validation using SCLC cell lines, organoids, or patient-derived models is required to determine whether the Risk Score is associated with actual drug response. ARRDC3 showed potential protective functional relevance *in vitro*. Draheim et al. (2010) demonstrated that ARRDC3 suppresses breast cancer progression by negatively regulating integrin β4, whereas Arakaki et al. (2018) reported that ARRDC3 inhibits breast carcinoma invasion by regulating G protein-coupled receptor lysosomal sorting and signaling. Consistent with these findings, ARRDC3 overexpression inhibited proliferation, colony formation, migration, and invasion in H446 cells, supporting its potential functional relevance as a key model-associated gene in SCLC. However, the current experiments did not directly assess CMA flux or demonstrate that ARRDC3 regulates CMA activity. Therefore, ARRDC3 should be interpreted as a CMA-related model-associated gene with functional relevance to malignant phenotypes, rather than as a confirmed CMA regulator.

Several limitations should be acknowledged. First, this study was mainly based on publicly available transcriptomic datasets. Although multiple cohorts were included and cross-cohort evaluation was performed, heterogeneity arising from different platforms, sample sources, and clinical backgrounds could not be completely eliminated. Second, available SCLC bulk transcriptomic cohorts with complete clinical annotations remain limited, and missing clinical variables may have affected further evaluation of the relationship between the model and clinical staging. Third, drug sensitivity analysis was mainly based on transcriptome-based predictive modeling and requires further validation using SCLC cell lines, organoids, or patient-derived models. In addition, *in vitro* validation was primarily performed in a single SCLC cell line, H446, and the current experiments were based on ARRDC3 overexpression. Therefore, these findings should be interpreted as preliminary evidence for the functional relevance of ARRDC3. Further loss-of-function experiments, rescue assays, and validation in additional SCLC cell lines or subtype-specific models are needed to confirm the functional role of ARRDC3 across different SCLC molecular contexts. Despite these limitations, this study integrates single-cell-level treatment response-associated heterogeneity, bulk-level CMA co-expression features, cross-cohort machine learning modeling, functional and immune characterization, drug sensitivity prediction, and *in vitro* validation into a relatively comprehensive evidence framework. These findings provide new insights into CMA-related cellular states, resistance evolution, and potential therapeutic strategies in SCLC.

## 5 Conclusion

This study integrated single-cell transcriptomics, bulk transcriptomics, and machine learning analysis to construct a CMA-related prognostic model for SCLC. The model effectively stratified patients into high-risk and low-risk groups and was associated with proliferation, cell-cycle progression, metabolism, immune microenvironment features, and drug sensitivity patterns. *In vitro* experiments further supported the functional role of ARRDC3 in regulating malignant phenotypes of SCLC cells. This study provides a new reference for molecular risk stratification and CMA-related resistance research in SCLC.

## Data Availability

The original contributions presented in the study are included in the article/supplementary material, further inquiries can be directed to the corresponding author.

## References

[B1] Arakaki A. K. S. Pan W. A. Lin H. Trejo J. (2018). The α-arrestin ARRDC3 suppresses breast carcinoma invasion by regulating G protein-coupled receptor lysosomal sorting and signaling. J. Biol. Chem. 293 (9), 3350–3362. 10.1074/jbc.RA117.001516 29348172 PMC5836114

[B2] Ashton T. M. McKenna W. G. Kunz-Schughart L. A. Higgins G. S. (2018). Oxidative phosphorylation as an emerging target in cancer therapy. Clin. Cancer Res. 24 (11), 2482–2490. 10.1158/1078-0432.CCR-17-3070 29420223

[B3] Baine M. K. Hsieh M.-S. Lai W. V. Egger J. V. Jungbluth A. A. Daneshbod Y. (2020). SCLC subtypes defined by ASCL1, NEUROD1, POU2F3, and YAP1: a comprehensive immunohistochemical and histopathologic characterization. J. Thorac. Oncol. 15 (12), 1823–1835. 10.1016/j.jtho.2020.09.009 33011388 PMC8362797

[B4] Caliman E. Fancelli S. Petroni G. Gatta Michelet M. R. Cosso F. Ottanelli C. (2023). Challenges in the treatment of small cell lung cancer in the era of immunotherapy and molecular classification. Lung Cancer 175, 88–100. 10.1016/j.lungcan.2022.11.014 36493578

[B5] Chan J. M. Quintanal-Villalonga Á. Gao V. R. Xie Y. Allaj V. Chaudhary O. (2021). Signatures of plasticity, metastasis, and immunosuppression in an atlas of human small cell lung cancer. Cancer Cell 39 (11), 1479–1496.e18. 10.1016/j.ccell.2021.09.008 34653364 PMC8628860

[B6] Chen X. Shi C. He M. Xiong S. Xia X. (2023). Endoplasmic reticulum stress: molecular mechanism and therapeutic targets. Signal Transduct. Target. Ther. 8 (1), 352. 10.1038/s41392-023-01570-w 37709773 PMC10502142

[B7] Chen Y. Li H. Fan Y. (2023). Shaping the tumor immune microenvironment of SCLC: mechanisms, and opportunities for immunotherapy. Cancer Treat. Rev. 120, 102606. 10.1016/j.ctrv.2023.102606 37579532

[B8] Cuervo A. M. Dice J. F. (1996). A receptor for the selective uptake and degradation of proteins by lysosomes. Science 273 (5274), 501–503. 10.1126/science.273.5274.501 8662539

[B9] Dice J. F. (1990). Peptide sequences that target cytosolic proteins for lysosomal proteolysis. Trends Biochem. Sci. 15 (8), 305–309. 10.1016/0968-0004(90)90019-8 2204156

[B10] Draheim K. M. Chen H. B. Tao Q. Moore N. Roche M. Lyle S. (2010). ARRDC3 suppresses breast cancer progression by negatively regulating integrin beta4. Oncogene 29 (36), 5032–5047. 10.1038/onc.2010.250 20603614 PMC2997682

[B11] Feng Q. Huang Z. Song L. Wang L. Lu H. Wu L. (2023). Combining bulk and single-cell RNA-Sequencing data to develop an NK cell-related prognostic signature for hepatocellular carcinoma based on an integrated machine learning framework. Eur. J. Med. Res. 28 (1), 306. 10.1186/s40001-023-01300-6 37649103 PMC10466881

[B12] Ganti A. K. P. Loo B. W. Bassetti M. Blakely C. Chiang A. D'Amico T. A. (2021). Small cell lung cancer, version 2.2022, NCCN Clinical Practice Guidelines in oncology. J. Natl. Compr. Canc Netw. 19 (12), 1441–1464. 10.6004/jnccn.2021.0058 34902832 PMC10203822

[B13] Gay C. M. Stewart C. A. Park E. M. Diao L. Groves S. M. Heeke S. (2021). Patterns of transcription factor programs and immune pathway activation define four major subtypes of SCLC with distinct therapeutic vulnerabilities. Cancer Cell 39 (3), 346–360.e7. 10.1016/j.ccell.2020.12.014 33482121 PMC8143037

[B14] Gazdar A. F. Bunn P. A. Minna J. D. (2017). Small-cell lung cancer: what we know, what we need to know and the path forward. Nat. Rev. Cancer 17 (12), 725–737. 10.1038/nrc.2017.106 29077690

[B15] George J. Lim J. S. Jang S. J. Cun Y. Ozretić L. Kong G. (2015). Comprehensive genomic profiles of small cell lung cancer. Nature 524 (7563), 47–53. 10.1038/nature14664 26168399 PMC4861069

[B16] Horn L. Mansfield A. S. Szczęsna A. Havel L. Krzakowski M. Hochmair M. J. (2018). First-line atezolizumab plus chemotherapy in extensive-stage small-cell lung cancer. N. Engl. J. Med. 379 (23), 2220–2229. 10.1056/nejmoa1809064 30280641

[B17] Hosaka Y. Araya J. Fujita Y. Kuwano K. (2021). Role of chaperone-mediated autophagy in the pathophysiology including pulmonary disorders. Inflamm. Regen. 41 (1), 29. 10.1186/s41232-021-00180-9 34593046 PMC8485456

[B18] Ichikawa A. Fujita Y. Hosaka Y. Kadota T. Ito A. Yagishita S. (2020). Chaperone-mediated autophagy receptor modulates tumor growth and chemoresistance in non-small cell lung cancer. Cancer Sci. 111 (11), 4154–4165. 10.1111/cas.14629 32860290 PMC7648026

[B19] Ireland A. S. Micinski A. M. Kastner D. W. Guo B. Wait S. J. Spainhower K. B. (2020). MYC drives temporal evolution of small cell lung cancer subtypes by reprogramming neuroendocrine fate. Cancer Cell 38 (1), 60–78.e12. 10.1016/j.ccell.2020.05.001 32473656 PMC7393942

[B20] Johnson W. E. Li C. Rabinovic A. (2007). Adjusting batch effects in microarray expression data using empirical Bayes methods. Biostatistics 8 (1), 118–127. 10.1093/biostatistics/kxj037 16632515

[B21] Kaushik S. Cuervo A. M. (2018). The coming of age of chaperone-mediated autophagy. Nat. Rev. Mol. Cell Biol. 19 (6), 365–381. 10.1038/s41580-018-0001-6 29626215 PMC6399518

[B22] Khawaja R. R. Martín-Segura A. Santiago-Fernández O. Sereda R. Lindenau K. McCabe M. (2025). Sex-specific and cell-type-specific changes in chaperone-mediated autophagy across tissues during aging. Nat. Aging 5 (4), 691–708. 10.1038/s43587-024-00799-6 39910244 PMC12003181

[B23] Korsunsky I. Millard N. Fan J. Slowikowski K. Zhang F. Wei K. (2019). Fast, sensitive and accurate integration of single-cell data with Harmony. Nat. Methods 16 (12), 1289–1296. 10.1038/s41592-019-0619-0 31740819 PMC6884693

[B24] Kuksin M. Morel D. Aglave M. Danlos F.-X. Marabelle A. Zinovyev A. (2021). Applications of single-cell and bulk RNA sequencing in onco-immunology. Eur. J. Cancer 149, 193–210. 10.1016/j.ejca.2021.03.005 33866228

[B25] Langfelder P. Horvath S. (2008). WGCNA: an R package for weighted correlation network analysis. BMC Bioinforma. 9, 559. 10.1186/1471-2105-9-559 19114008 PMC2631488

[B26] Liu S. V. Reck M. Mansfield A. S. Mok T. Scherpereel A. Reinmuth N. (2021). Updated overall survival and PD-L1 subgroup analysis of patients with extensive-stage small-cell lung cancer treated with atezolizumab, carboplatin, and etoposide (IMpower133). J. Clin. Oncol. 39 (6), 619–630. 10.1200/jco.20.01055 33439693 PMC8078320

[B27] Liu J. Wang L. He H. Liu Y. Jiang Y. Yang J. (2023). The complex role of chaperone-mediated autophagy in cancer diseases. Biomedicines 11 (7), 2050. 10.3390/biomedicines11072050 37509689 PMC10377530

[B28] Megyesfalvi Z. Barany N. Lantos A. Valko Z. Pipek O. Lang C. (2022). Expression patterns and prognostic relevance of subtype-specific transcription factors in surgically resected small-cell lung cancer: an international multicenter study. J. Pathol. 257 (5), 674–686. 10.1002/path.5922 35489038 PMC9541929

[B29] Mollaoglu G. Guthrie M. R. Böhm S. Brägelmann J. Can I. Ballieu P. M. (2017). MYC drives progression of small cell lung cancer to a variant neuroendocrine subtype with vulnerability to Aurora kinase inhibition. Cancer Cell 31 (2), 270–285. 10.1016/j.ccell.2016.12.005 28089889 PMC5310991

[B30] Paz-Ares L. Dvorkin M. Chen Y. Reinmuth N. Hotta K. Trukhin D. (2019). Durvalumab plus platinum–etoposide versus platinum–etoposide in first-line treatment of extensive-stage small-cell lung cancer (CASPIAN): a randomised, controlled, open-label, phase 3 trial. Lancet 394 (10212), 1929–1939. 10.1016/s0140-6736(19)32222-6 31590988

[B31] Pudova E. A. Pavlov V. S. Guvatova Z. G. Fedorova M. S. Shegai P. V. Kudryavtseva A. V. (2025). Machine learning models for cancer research: a narrative review of bulk RNA-seq applications. Int. J. Mol. Sci. 26 (24), 12081. 10.3390/ijms262412081 41465507 PMC12732975

[B32] Qi J. Zhang J. Liu N. Zhao L. Xu B. (2022). Prognostic implications of molecular subtypes in primary small cell lung cancer and their correlation with cancer immunity. Front. Oncol. 12, 779276. 10.3389/fonc.2022.779276 35311069 PMC8924463

[B33] Ritchie M. E. Phipson B. Wu D. Hu Y. F. Law C. W. Shi W. (2015). Limma powers differential expression analyses for RNA-sequencing and microarray studies. Nucleic Acids Res. 43 (7), e47. 10.1093/nar/gkv007 25605792 PMC4402510

[B34] Rudin C. M. Brambilla E. Faivre-Finn C. Sage J. (2021). Small-cell lung cancer. Nat. Rev. Dis. Prim. 7 (1), 3. 10.1038/s41572-020-00235-0 33446664 PMC8177722

[B35] Sebaugh J. L. (2011). Guidelines for accurate EC50/IC50 estimation. Pharm. Stat. 10 (2), 128–134. 10.1002/pst.426 22328315

[B36] Sen T. Gay C. M. Byers L. A. (2018). Targeting DNA damage repair in small cell lung cancer and the biomarker landscape. Transl. Lung Cancer Res. 7 (1), 50–68. 10.21037/tlcr.2018.02.03 29535912 PMC5835589

[B37] Shi Z.-D. Pang K. Wu Z.-X. Dong Y. Hao L. Qin J.-X. (2023). Tumor cell plasticity in targeted therapy-induced resistance: mechanisms and new strategies. Signal Transduct. Target. Ther. 8 (1), 113. 10.1038/s41392-023-01383-x 36906600 PMC10008648

[B38] Stuart T. Butler A. Hoffman P. Hafemeister C. Papalexi E. Mauck W. M. (2019). Comprehensive integration of single-cell data. Cell 177 (7), 1888–1902.e21. 10.1016/j.cell.2019.05.031 31178118 PMC6687398

[B39] Teixeira A. Ramalho M. C. C. Souza I. Andrade I. A. M. Osawa I. Y. A. Guedes C. B. (2024). The role of chaperone-mediated autophagy in drug resistance. Genet. Mol. Biol. 47 (1), e20230317. 10.1590/1678-4685-gmb-2023-0317 38829285 PMC11145944

[B40] Tian Y. Li Q. Yang Z. Zhang S. Xu J. Wang Z. (2022). Single-cell transcriptomic profiling reveals the tumor heterogeneity of small-cell lung cancer. Signal Transduct. Target. Ther. 7 (1), 346. 10.1038/s41392-022-01150-4 36195615 PMC9532437

[B41] Uslu C. Kapan E. Lyakhovich A. (2024). Cancer resistance and metastasis are maintained through oxidative phosphorylation. Cancer Lett. 587, 216705. 10.1016/j.canlet.2024.216705 38373691

[B42] Wang Y. Liu J. Wang H. Jiang P. Cao L. Lu S. (2025). Multiple regulatory mechanisms, functions and therapeutic potential of chaperone-mediated autophagy. Theranostics 15 (7), 2778–2793. 10.7150/thno.107761 40083922 PMC11898275

[B43] Wu T. Z. Hu E. Q. Xu S. B. Chen M. J. Guo P. F. Dai Z. H. (2021). clusterProfiler 4.0: a universal enrichment tool for interpreting omics data. Innovation 2 (3), 100141. 10.1016/j.xinn.2021.100141 34557778 PMC8454663

[B44] Xie S. Hou S. Chen J. Qi X. (2025). Integrative single-cell and bulk RNA sequencing identifies a macrophage-related prognostic signature for predicting prognosis and therapy responses in colorectal cancer. Int. J. Mol. Sci. 26 (2), 811. 10.3390/ijms26020811 39859524 PMC11765994

[B45] Yoshihara K. Shahmoradgoli M. Martínez E. Vegesna R. Kim H. Torres-Garcia W. (2013). Inferring tumour purity and stromal and immune cell admixture from expression data. Nat. Commun. 4, 2612. 10.1038/ncomms3612 24113773 PMC3826632

[B46] Ysebaert L. Quillet-Mary A. Tosolini M. Pont F. Laurent C. Fournié J.-J. (2021). Lymphoma heterogeneity unraveled by single-cell transcriptomics. Front. Immunol. 12, 597651. 10.3389/fimmu.2021.597651 33732232 PMC7959738

[B47] Zhang C. Li J. Tang Q. Li L. Cao D. (2025). Targeting proteostasis for cancer therapy: current advances, challenges, and future perspectives. Mol. Cancer 24 (1), 265. 10.1186/s12943-025-02472-x 41121138 PMC12542412

